# Pharmacological advances in multi-targeted strategies for type 2 diabetes mellitus: a systematic perspective based on traditional Chinese medicine

**DOI:** 10.3389/fphar.2025.1732134

**Published:** 2026-02-20

**Authors:** Yan-Li Zhao, Jia-Bao Liao, Pan-Pan Pang, Jing-Yuan Li, Suo-Cai Su, Meng-Qiu Shao, Wei-Bo Wen, Fu-Rong Xu

**Affiliations:** 1 School of the First Clinical Medical College, Yunnan University of Chinese Medicine, Kunming, Yunnan, China; 2 School of Pharmaceutical Science and Yunnan Key Laboratory of Pharmacology for Natural Products, Kunming Medical University, Kunming, China; 3 School of Chinese Medicine, Yunnan University of Chinese Medicine, Kunming, Yunnan, China

**Keywords:** botanical drugs, botanical preparations, diabetic complications, evidence grading, multi-omics, network pharmacology, plant metabolites, systems pharmacology

## Abstract

Type 2 diabetes mellitus (T2DM) is a complex systemic metabolic disease driven by insulin resistance, β-cell dysfunction, chronic low-grade inflammation, oxidative stress, and neuro-immune dysregulation. It frequently progresses to multi-organ complications affecting the kidneys, retina, heart, and central nervous system. This review synthesizes mechanistic and translational evidence on Traditional Chinese Medicine (TCM)-related botanical drugs and botanical preparations (formula-based interventions), along with representative plant metabolites that are frequently investigated in the TCM research context (e.g., berberine, baicalin, and tanshinone IIA, which are not unique to TCM). For formula-based preparations, we extracted and reported intervention identity elements (dosage form, complete composition, and processing/standardization as described in primary studies); missing identity items were recorded as not reported (NR) and not inferred. We organized findings across shared T2DM-relevant pathogenic modules, including PI3K/Akt and AMPK signaling, inflammatory outputs (NF-κB/NLRP3), redox regulation (NRF2/ROS), angiogenic signaling (VEGF), and gut–liver–brain–immune network interactions, emphasizing studies in which pathway modulation is accompanied by metabolic or complication-relevant endpoints. To strengthen interpretability and reproducibility, we conducted a structured literature search (2000–2025) and applied evidence grading (human/RCT vs. animal vs. in vitro/in silico), and we critically appraised reporting quality using the GA-online Best Practice in Research – ConPhyMP tool. All source organisms were taxonomically validated using authoritative resources, and full scientific names (including author citation and family) were standardized. We caution that compound–target links, particularly those derived from in silico predictions or single-assay readouts, may be vulnerable to assay interference liabilities (including PAINS) and should be supported by orthogonal validation and outcome-linked readouts before strong mechanistic claims are made. Finally, we outline translational priorities, including rigorous standardization and quality control (distinguishing analytical marker metabolites from bioactive metabolites), improved study design and controls, and well-designed randomized, pragmatic, and real-world evaluations with clinically meaningful endpoints (e.g., HbA1c, complication progression, and safety).

## 1 Introduction

Type 2 diabetes mellitus (T2DM) is a complex systemic metabolic disease characterized by insulin resistance, β-cell dysfunction, chronic low-grade inflammation, and oxidative stress (Butler et al., 2003; Samuel and Shulman, 2012; Du et al., 2003; Defronzo, 2009; Fu et al., 2021; Goldfine et al., 2013; Ryuk et al., 2017; Shi et al., 2019; Peng et al., 2021). According to the International Diabetes Federation (IDF) Diabetes Atlas (2025), an estimated 589 million adults (20–79 years) were living with diabetes worldwide in 2024 (≈1 in 9), and the total number is projected to rise to 853 million by 2050 (≈1 in 8) (International Diabetes Federation, 2025; GBD 2021 Diabetes Collaborators, 2023; An et al., 2023). According to the International Diabetes Federation (IDF) Diabetes Atlas (11th edition), China has the highest number of adults (20–79 years) living with diabetes, with an estimated adult prevalence of approximately 11.9% by 2024. Notably, prevalence estimates can vary by data source and diagnostic criteria; for example, a large nationally representative survey in China reported an overall diabetes prevalence of 12.4% in 2018 (Li et al., 2017; Wang L et al., 2021). In addition to chronic hyperglycemia, T2DM frequently manifests with cardiovascular and microvascular complications (e.g., diabetic kidney disease, neuropathy, and retinopathy), collectively forming a complex and interconnected pathological network (Luo et al., 2009). Contemporary guideline-recommended therapies for T2DM, including metformin and agents with proven cardio–renal benefits, such as sodium–glucose cotransporter 2 (SGLT2) inhibitors and glucagon-like peptide-1 (GLP-1) receptor agonists, can improve glycemic control and reduce major cardiovascular and/or kidney outcomes. However, residual risk and incomplete prevention of complication progression remain in many patients, underscoring the need for complementary strategies that target broader pathogenic modules beyond glycemia. In this context, TCM-related multi-botanical preparations (formula-based botanical preparations), which comprise multiple constituents and diverse plant metabolites, may offer a complementary, formula-centered framework to co-modulate overlapping disease-relevant modules (e.g., inflammation, oxidative stress, insulin signaling, and neuroendocrine regulation), provided that botanical drug identity, preparation standardization/quality control, dosing ranges, appropriate controls, and outcome-linked endpoints are transparently reported and evidence graded (Yao et al., 2024). Accordingly, for each multi-botanical preparation (including classical formulas such as Jin-Gui Shen-Qi Wan [Jin Gui Shen Qi Pill]), we extracted and reported intervention identity unambiguously, including dosage form, complete composition, and processing/extraction/standardization, as described in the primary studies. Missing identity items were recorded as NR (not reported) and were not inferred. Studies have shown that the bioactive plant metabolites of TCM-related botanical drugs cooperatively regulate key metabolic and inflammatory signaling pathways, including PI3K/Akt, AMPK, NF-κB, and NLRP3, influencing the signaling interactions within the gut-liver-brain–immune axis, thereby exerting systemic regulatory effects (Ng et al., 2024; Wu et al., 2025; Ghafouri-Fard et al., 2022; Lin et al., 2023; Wang et al., 2025).

**FIGURE 1:**
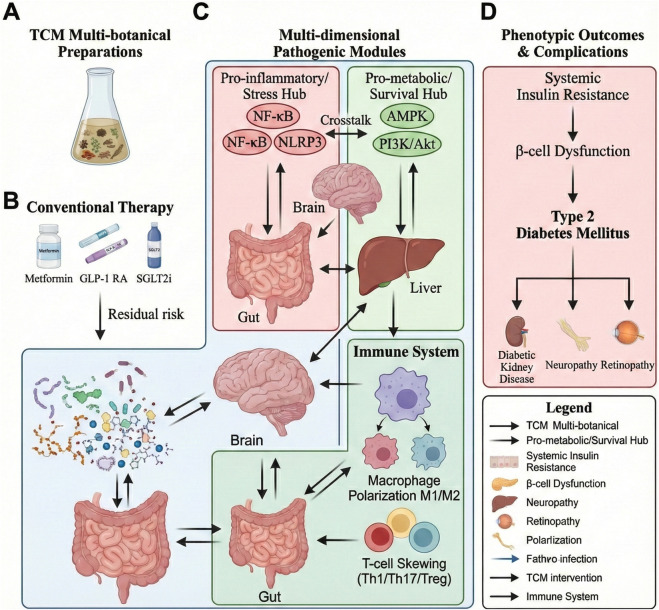
Systems-level framework linking TCM interventions to the gut–liver–brain–immune axis in T2DM. **(A)** Multi-botanical, formula-based TCM preparations provide diverse bioactive candidates with standardization and quality control. **(B)** Guideline therapies (metformin, GLP-1 RA, SGLT2i) improve glycemia; however, residual risk may persist. **(C)** Gut–liver–brain–immune crosstalk converges on a pro-inflammatory/stress hub (NF-κB–linked transcription, NLRP3 inflammasome) and a pro-metabolic/survival hub (AMPK, PI3K/Akt), with immune imbalance (macrophage polarization, T-cell skewing) reinforcing chronic low-grade inflammation. **(D)** These interactions drive insulin resistance, β-cell dysfunction, and other major complications. Line semantics: solid, direct regulation; dashed, indirect associations; red, pathological promotion; green, therapeutic restoration; blunt-ended, inhibition.

### 1.1 Methods: literature search, study selection, and critical appraisal

We conducted a structured literature search to identify preclinical and clinical studies evaluating Traditional Chinese Medicine (TCM) formulas, botanical extracts, and representative plant metabolites relevant to type 2 diabetes mellitus (T2DM) and its major complications. Searches were performed in PubMed/MEDLINE, Web of Science, and Scopus from January 2000 to December 2025 using combinations of controlled vocabularies and free text terms. The core PubMed search string was: (“type 2 diabetes” OR T2DM OR “diabetic complications”) AND (“traditional Chinese medicine” OR TCM OR “Chinese herbal” OR decoction OR formula OR “herbal extract”) AND (trial OR randomized OR RCT OR clinical OR preclinical OR *in vivo* OR *in vitro* OR “network pharmacology” OR “systems pharmacology” OR multi-omics), with additional compound/formula terms (e.g., berberine, baicalin, curcumin, Gegen Qinlian Decoction, Huanglian Jiedu Decoction, Jin-Gui Shen-Qi Wan [Jin Gui Shen Qi Pill]) used where appropriate. The reference lists of relevant reviews were screened to identify additional eligible studies. Titles and abstracts were screened first, followed by a full-text assessment. We included studies that (i) investigated a defined TCM formula/extract or a clearly specified phytochemical, (ii) used a T2DM-relevant model or patient population, and (iii) reported metabolic outcomes and/or complication-related endpoints. We excluded articles lacking sufficient methodological detail to evaluate the central pharmacological/clinical claims, studies with unclear intervention identity/standardization, and purely speculative discussions without primary data. The evidence was stratified into tiers (human/RCT, animal, and *in vitro*/*in silico*). For pharmacological studies, we extracted the model type, dose range and/or minimal active concentration (where reported), exposure duration, extract/compound characterization, and presence of appropriate positive/negative controls. For clinical studies, we extracted the study design, sample size, intervention standardization, concomitant therapies, clinically meaningful endpoints (e.g., HbA1c and complication-related outcomes), safety reporting, and follow-up duration. Where key information (e.g., dose–response/MAC, controls, extract characterization, composition and processing/standardization, or taxonomic validation) was not reported in the primary study, we did not infer missing details and instead recorded these items as NR (not reported) and considered them in the limitations appraisal of the study. All source organisms were taxonomically validated using authoritative resources (e.g., Kew MPNS and/or Plants of the World Online), and full scientific names including author citation and family (e.g., “*Salvia*”) are provided in the Supplementary Tables/Supplementary Material. To ensure a rigorous appraisal of the phytopharmacological evidence, we assessed the reporting quality and extract characterization using the GA-online Best Practice in Research–ConPhyMP tool (Supplementary Tables 1, 2A; sections relevant to reviews), provided as Supplementary File S2 (Heinrich et al., 2022; GA-online, 2025). The reporting is summarized in Supplementary Tables S1, S2, and a PRISMA-style study selection flow diagram is provided in Supplementary Figure S1.

## 2 Targeted modulation of key signaling pathways by TCM in T2DM

**FIGURE 2:**
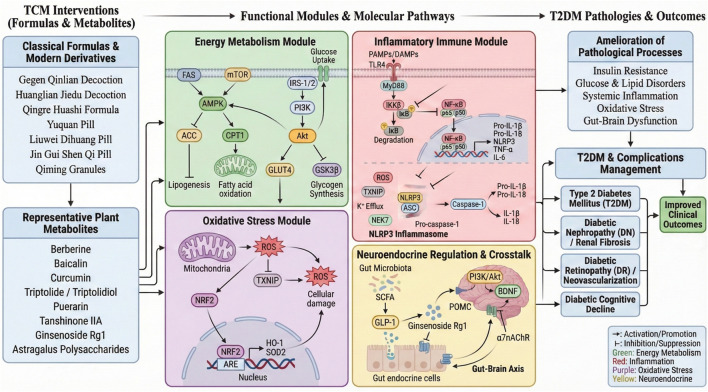
Systems pharmacology map linking TCM formulas/metabolites to functional modules and T2DM outcomes. Classical formulas and modern derivatives (left) provide representative metabolites that act on four interconnected modules: energy metabolism (AMPK–PI3K/Akt–GLUT4), inflammatory immunity (TLR4–MyD88–NF-κB and NLRP3 inflammasome), oxidative stress (NRF2–ARE/HO-1 axis), and neuroendocrine crosstalk (gut microbiota–SCFA/GLP-1 and α7nAChR–gut–brain signaling). The coordinated modulation of these modules ameliorates insulin resistance, metabolic dysregulation, systemic inflammation, oxidative stress, and gut–brain dysfunction, thereby improving T2DM and major complications (DN, DR, and cognitive decline). Arrows indicate activation/promotion or inhibition/suppression, and colors denote module categories.

The pathogenesis of T2DM involves multiple tightly coupled processes governed by core signaling modules (e.g., PI3K/Akt, AMPK, NF-κB, and NLRP3). Rather than implying a strict “single-target vs. multi-target” dichotomy, we used a systems-pharmacology lens to organize evidence on botanical formulas and constituents across shared T2DM-relevant modules, prioritizing studies in which pathway modulation is accompanied by metabolic or complication-related endpoints (e.g., HbA1c, HOMA-IR, and tissue injury markers) (Figure 2).

### 2.1 Systematic pharmacological explanation of TCM intervention in T2DM

While multiple studies indicate that TCM-related botanical drugs and botanical preparations modulate key signaling pathways such as PI3K/Akt, AMPK, NF-κB, and NRF2, these findings are often fragmented and may over-emphasize isolated target “hits.” To reduce over-interpretation, we explicitly distinguished evidence tiers (human/RCT vs. animal vs. *in vitro*/*in silico*) and interpreted compound–target claims cautiously because some plant metabolites may exhibit assay-interference liabilities (including PAINS) or non-specific effects; whenever possible, conclusions should rely on orthogonal validation and outcome-linked readouts rather than single-assay target engagement (Baell and Holloway, 2010; Dahlin et al., 2021). In this section, we use a systems-pharmacology lens to organize the evidence into multilayer networks linking formulas–plant metabolites–targets–pathways, and then map the targets into higher-order functional modules (e.g., energy metabolism, immune–inflammatory regulation, oxidative stress, and neuroendocrine regulation). By applying pathway enrichment (e.g., KEGG/GO), network clustering, and pathway mapping, the regulatory effects of TCM-related interventions can be aligned with core pathogenic characteristics of T2DM, thereby advancing from “point-target regulation” to “system module intervention.” Notably, many representative metabolites summarized in Table 1 (e.g., berberine, baicalin, and curcumin) are widely distributed natural products and are not unique to TCM; they are included here as exemplars frequently studied in the TCM research context to support ingredient–target–module mapping. Importantly, multi-target pharmacology is also observed in conventional single-molecule drugs (e.g., metformin). Here, we used a system pharmacology framework primarily to structure and compare formula-centered evidence into functional modules for T2DM (Table 1).

**TABLE 1:** Systems pharmacology functional-module mapping of TCM interventions in T2DM (ConPhyMP-aligned reporting additions in bold).

Functional module	Key pathways or targets	Representative TCM plant metabolites	Representative formulas	Clinical evidence tier	T2DM processes
Energy metabolism module (Zou et al., 2009)	AMPK, PPARγ, SIRT1	Berberine (from Coptis chinensis Franch. [Ranunculaceae]), Astragalus polysaccharides (from Astragalus membranaceus (Fisch.) Bunge [Fabaceae]/Astragalus mongholicus Bunge [Fabaceae], as reported)	Huanglian Jiedu Decoction (multi-botanical decoction; composition/processing/QC markers in Supplementary Table S1; NR if not reported), Gegen Qinlian Decoction (multi-botanical decoction; composition/processing/QC markers in Supplementary Table S1; NR if not reported)	Supported by clinical trials and animal studies	Insulin resistance, energy imbalance
Inflammatory immune module (Bai et al., 2021)	NF-κB, NLRP3, TNF-α, IL-6	Baicalin (from Scutellaria baicalensis Georgi [Lamiaceae]), Triptolide (from Tripterygium wilfordii Hook.f. [Celastraceae]), Curcumin (from Curcuma longa L. [Zingiberaceae])	Qingre Huashi Formula (multi-botanical preparation; composition/processing/QC markers in Supplementary Table S1; NR if not reported), Gegen Qinlian Decoction (multi-botanical decoction; composition/processing/QC markers in Supplementary Table S1; NR if not reported)	Supported by animal and *in vitro* studies	Chronic inflammation, immune dysregulation
Oxidative stress module (Chen et al., 2020; Bai F et al., 2021)	NRF2/HO-1, ROS, SOD	Tanshinone IIA (from Salvia miltiorrhiza Bunge [Lamiaceae]), Puerarin (primarily associated with Pueraria lobata (Willd.) Ohwi [Fabaceae], as reported)	Qiming Granules (Chinese patent medicine; full botanical composition/manufacturing and QC information in Supplementary Table S1; NR if not reported)	Supported mainly by animal/*in vitro* studies	Oxidative stress injury, diabetic complications
Neuroendocrine regulation module (Lei et al., 2023)	GLP-1, insulin receptor, BDNF	Ginsenoside Rg1 (from Panax ginseng C.A.Mey. [Araliaceae]), Poria polysaccharides (from Wolfiporia extensa (Peck) Ginns [Polyporaceae]; syn. Poria cocos, as reported)	Yuquan Pill (multi-botanical pill; composition/processing/QC markers in Supplementary Table S1; NR if not reported), Liuwei Dihuang Pills (multi-botanical pill; composition/processing/QC markers in Supplementary Table S1; NR if not reported)	Supported by some clinical trials	Insulin secretion, glucose control, cognitive decline
Metabolism–inflammation–neuro crosstalk module (integrative)	Cross-module integration	Combination of above metabolites (source species/authorities/families specified in respective rows; taxonomically validated; see Supplementary Table S1)	Huanglian Jiedu Decoction (composition/processing/QC markers in Supplementary Table S1; NR if not reported), Liuwei Dihuang Pills (composition/processing/QC markers in Supplementary Table S1; NR if not reported)	Supported by integrated multi-omics studies	Systemic metabolic syndrome, multi-organ complications

Botanical source species are taxonomically validated; full species names with authorities and family, plant part, and voucher information (if reported) are provided in Supplementary Table S1; items not reported in the primary study are recorded as **NR**. ‡ For multi-botanical preparations, the complete composition (all component drugs with validated species + authority + family), processing/preparation (e.g., decoction/granule/pill; extraction/solvent and ratio), and chemical characterization/QC markers are reported in Supplementary Table S1.

### 2.2 Interpreting classical formula compatibility (Jun–Chen–Zuo–Shi) within a multi-target network-module framework

In classical formula design, the “Jun–Chen–Zuo–Shi” (monarch–minister–assistant–courier) hierarchy provides a principled rationale for herb compatibility and dose allocation. The Jun (monarch) directly targets the core pathogenesis and contributes the primary therapeutic force; the Chen (minister) reinforces the monarch and/or addresses key co-pathologies; the Zuo (assistant) supports the main actions, moderates toxicity or excessive properties, and may treat accompanying symptoms; and the Shi (courier) harmonizes the formula and may guide actions to specific meridians, organs, or functional axes. This framework is widely used to structure multi-herb prescriptions and explain why the entire formula can outperform individual constituents in complex diseases (Luan et al., 2020; Wu et al., 2014). Mechanistically, the hierarchy can be mapped to multi-target regulation: monarchs/ministers tend to cover high-centrality nodes and dominant pathways, whereas assistants/couriers fine-tune network topology, pharmacokinetics, and tissue distribution, thereby improving the efficacy–safety balance of the drug. Recent systems approaches, such as network pharmacology and multi-omics integration, provide quantitative tools to test such role assignments by linking herb-derived compounds to targets, pathways, and phenotypes at the module level. These approaches align with the broader concept of network pharmacology, in which polypharmacology is leveraged to modulate disease networks rather than isolated targets (Hopkins, 2008; Wu et al., 2014; Zhou et al., 2016). Therefore, making the compatibility principle explicit in anti-T2DM formulas helps readers interpret how “multi-component–multi-target” actions are organized, facilitates more reproducible mechanism annotation across studies, and provides clearer mapping between mechanistic modules and clinical outcomes (Luan et al., 2020; Cheng et al., 2017).

### 2.3 Major anti-T2DM classical formula lineages and their modernization derivatives

To address the potential incompleteness of the formula coverage, we expanded the anti-T2DM TCM formula section by adding a lineage-oriented summary table (Table 2). The table includes representative classical core prescriptions and their modern derivatives/commercial Chinese polyherbal preparation (CCPP) that are frequently discussed in contemporary clinical literature, spanning major TCM pattern types relevant to T2DM (e.g., heat, dampness, qi–yin deficiency, and kidney deficiency) and key complication domains (e.g., diabetic nephropathy and retinopathy). For each formula-based preparation listed in Table 2 (including proprietary/commercial Chinese polyherbal preparation (CCPP)), we report intervention identity unambiguously—dosage form, complete composition, and processing/standardization/quality control as described in the primary sources—and record missing items as NR (not reported) without inference, consistent with the GA-online Best Practice/ConPhyMP requirements. All source organisms for component botanical drugs were taxonomically validated using authoritative databases (e.g., Kew MPNS and/or Plants of the World Online), and full scientific names, including author citation and family, are provided in Table 2 and/or the accompanying identity table. We also indicated the typical mechanistic modules for each lineage to facilitate cross-study comparisons with the pathway framework in Table 1 (Tian et al., 2019; Tan et al., 2024; Hu et al., 2021a; Zhang et al., 2024).

**TABLE 2:** Major anti-T2DM classical formula lineages and representative modern derivatives (ConPhyMP aligned reporting signposting).

Lineage/Therapeutic principle	Representative classical formula (English name; abbreviation)	Common modern derivatives/Commercial Chinese polyherbal preparation (CCPP) (examples)	Typical mechanistic modules (examples)	Composition & processing/Taxonomic validation (required; where not reported, record as NR)**
Heat-clearing and damp-heat resolving	Gegen Qinlian Decoction (GQD)	GQD granules; related decoction derivatives used in trials	PI3K/Akt; AMPK; NF-κB/NLRP3; gut microbiota–SCFA	Provide full composition for GQD (all component drugs) with taxonomically validated source species (authority + family), plant part, and voucher specimen (if reported); specify preparation (decoction/granule), extraction solvent, drug:solvent ratio, concentration, dosing, and quantitative QC markers/fingerprint; otherwise mark NR.
Qi–Yin replenishing (Xiaoke-related)	Yuquan Pill (YQP)	Yuquan-based commercial Chinese polyherbal preparation (CCPP) (where applicable)	AMPK; PI3K/Akt; oxidative-stress modules; gut–endocrine relay (reported)	For YQP/patent derivatives: report manufacturer/pharmacopeial reference (if applicable), dosage form, complete composition with validated species (authority + family), processing (Paozhi), and quantitative QC markers; otherwise mark NR.
Yin-nourishing and deficiency-heat clearing	Liuwei Dihuang Pill (LDP; LWDHW)	LWDHW-based derivatives (e.g., modified pills/granules in trials)	Gut microbiota–SCFA–GLP-1 (reported); anti-inflammatory and redox modules	Report complete composition for LWDHW-based interventions with validated species (authority + family), plant part, voucher (if reported), and preparation/QC details (dosage form, extraction/processing, marker quantification, batch consistency); otherwise mark NR.
Kidney-supporting (chronic deficiency patterns)	Jin Gui Shen Qi Wan (JGSQW; also known as Shenqi Wan)	JGSQW-based pills/granules; related “kidney-supporting” derivatives	PI3K/Akt; AMPK; redox/inflammation modules (reported)	Define the preparation unambiguously (JGSQW/Shenqi Wan; classical formula source if stated in primary study) and report complete composition with validated species (authority + family), processing/preparation, dosage form, and quantitative QC markers; otherwise mark NR.
Complication-oriented derivatives (microvascular protection)	— (lineage includes formula-derived complication prescriptions)	Qiming Granules (DR adjunct); Huang Kui Capsules (DN); others as applicable	VEGF/HIF-1α; NRF2/ROS; TGF-β/SMAD; inflammatory modules	For multi-botanical proprietary preparations: report complete composition with validated species (authority + family), plant part, voucher (if reported), dosage form, manufacturer, preparation/extraction details, and quantitative QC markers/batch numbers; otherwise mark NR.

Botanical source species are taxonomically validated; full species names with authorities and family, plant part, and voucher information (if reported) are provided in Supplementary Table S1; items not reported in the primary study are recorded as NR. For multi-botanical preparations, complete composition (all component drugs with validated species + authority + family), processing/preparation (e.g., decoction/granule/pill; extraction/solvent and ratio), and chemical characterization/QC markers are reported in Supplementary Table S1. ** “NR” indicates not reported in the original study and was not inferred by the authors.

### 2.4 Activation of the PI3K/Akt pathway to enhance insulin sensitivity

The PI3K/Akt module is a core insulin signaling axis that supports glucose homeostasis by promoting GLUT4 membrane translocation in skeletal muscle/adipose tissue and restraining hepatic gluconeogenesis, thereby contributing to insulin sensitivity (Schultze et al., 2012; Huang et al., 2018). In T2DM, impaired signaling is commonly reflected by reduced Akt phosphorylation and attenuated GLUT4 translocation, which correlates with insulin resistance (Tonks et al., 2013). In this review, we discuss PI3K/Akt primarily as a T2DM-relevant functional module and prioritize studies in which PI3K/Akt modulation is accompanied by metabolic outcome-linked readouts (e.g., fasting glucose, HOMA-IR/insulin tolerance, and skeletal muscle glucose uptake markers) rather than pathway readouts alone. Preclinical studies have reported that plant metabolites or defined botanical preparations can engage this module; for example, polysaccharides from Astragali Radix (source species taxonomically validated; e.g., *Astragalus membranaceus* (Fisch.) Bunge [Fabaceae], as reported in the primary study), have been described to enhance insulin receptor substrate (IRS)–PI3K coupling and Akt activation in diabetes-relevant models, with concurrent improvements in insulin-sensitivity-related phenotypes (Kearney et al., 2021). Puerarin (an isoflavone primarily associated with *Pueraria lobata* (Willd.) Ohwi [Fabaceae], as reported) has been reported to attenuates JNK-associated inhibitory signaling and preserves IRS-1/2 signaling integrity, together with improved glucose handling in experimental diabetes settings (Huang et al., 2012). At the formula level, Gegen Qinlian Decoction (GQD) is a classical multi-herb decoction; its complete composition and processing/standardization (as reported; NR if not reported) are provided in Table 2/Supplementary Table S1 GQD has been associated with reduced SOCS3 expression and enhanced skeletal muscle glucose uptake with concurrent insulin resistance improvement in preclinical studies (Jorgensen et al., 2013). Nevertheless, because PI3K/Akt is pleiotropic and broadly involved in apoptosis, lipid metabolism, and inflammatory signaling, we interpret PI3K/Akt modulation as supportive mechanistic evidence and emphasize the need for dose-plausible designs, appropriate controls, and outcome-linked endpoints when translating these findings into T2DM management.

### 2.5 AMPK pathway: restoring cellular energy homeostasis

AMP-activated protein kinase (AMPK) is a central energy-sensing kinase that coordinates glycolysis, fatty acid oxidation, and mitochondrial function to maintain metabolic homeostasis (Herzig and Shaw, 2018). In T2DM, nutrient overload and impaired insulin signaling are commonly associated with reduced AMPK activity, which contributes to metabolic dysregulation. In this review, we discuss AMPK primarily as a T2DM-relevant functional module and prioritize studies in which AMPK activation is accompanied by metabolic outcome-linked readouts (e.g., fasting glucose, insulin sensitivity indices, and lipid accumulation markers) rather than AMPK phosphorylation alone. Preclinical studies have reported that representative plant metabolites investigated in the TCM research context can activate this pathway. For instance, berberine (a protoberberine alkaloid commonly associated with Coptidis Rhizoma; source species taxonomically validated and provided in the Supplementary Tables/Supplementary Material) promotes AMPK phosphorylation and increases GLUT4 expression and membrane translocation in diabetes-relevant experimental settings with concurrent improvements in insulin-resistance–related phenotypes (Lee et al., 2006). Tanshinone IIA (a diterpenoid quinone from Salvia) has been reported to attenuate lipogenesis via the AMPK–ACC axis and improve lipid-related readouts in experimental models (Gao et al., 2021). Astragalus glycosides (from Astragali Radix; source species taxonomically validated and provided in the Supplementary Tables/Supplementary Material, as reported) have been described to activate AMPK and PI3K/Akt signaling in animal studies, with a concomitant enhancement of glucose uptake and insulin sensitivity (Gong et al., 2023). At the formula level, Huanglian Jiedu Decoction (Huanglian-Jiedu Decoction; HLJDT) is a classical multi-herb decoction, and its complete composition and processing/standardization (as reported; NR if not reported) are provided in Table 2/Supplementary Table S1. It has been reported to modulate fatty acid utilization markers (e.g., upregulating CPT1 and suppressing fatty acid synthase), consistent with a shift toward improved energy metabolism in preclinical studies (Hu et al., 2021b). Nevertheless, because AMPK intersects multiple biological programs beyond glucose control, we interpret AMPK-related modulation as supportive mechanistic evidence and emphasize the need for dose-plausible designs (Wang M. et al., 2024), appropriate controls, and clinically meaningful endpoints when translating these findings into T2DM management strategies.

### 2.6 NF-κB pathway: inhibiting inflammation and preserving insulin signaling

The NF-κB program represents a convergent inflammatory transcriptional output that is chronically engaged in obesity/T2DM and is closely linked to insulin resistance and tissue injury in the metabolic organs. In T2DM, sustained inflammatory signaling in the adipose tissue, liver, skeletal muscle, and pancreatic islets can impair insulin action and exacerbate β-cell stress, making NF-κB a disease-relevant module, rather than a purely generic pathway. Importantly, clinical proof-of-concept exists that dampening upstream inflammatory signaling (including IKKβ/NF-κB–related activity) can improve glycemic control and inflammatory biomarkers in patients with T2DM (Goldfine et al., 2010; Goldfine and Shoelson, 2017). As evidence-linked examples from the literature on TCM-related botanical drugs and plant metabolites, baicalin (a flavone glycoside primarily associated with *Scutellaria baicalensis* Georgi [Lamiaceae], as reported) has been reported to suppress NF-κB activation (e.g., reduced IKKβ phosphorylation and p65 nuclear translocation) in models where improvements in inflammatory readouts co-occur with better metabolic phenotypes (Shen et al., 2019). Curcumin, a widely studied plant metabolite (primarily associated with *Curcuma longa* L. [Zingiberaceae], as reported) has been associated with reduced inflammatory signaling alongside antioxidant responses (e.g., NRF2-related markers) in diabetes-relevant settings (Ghareghomi et al., 2021). At the formula level, Qingre Huashi Formula, a botanical preparation, was treated as a formula-based intervention with explicitly extracted intervention identity (dosage form, complete composition, and processing/standardization as reported; NR if not reported), provided in Table 2/Supplementary Table S1, and has been reported to modulate upstream innate-immune signaling (e.g., TLR4/MyD88) with concurrent reductions in pro-inflammatory cytokines and improvements in insulin signaling or vascular-related readouts (Tian et al., 2021). Nevertheless, NF-κB is pleiotropic and widely involved in many biological processes; therefore, we interpret NF-κB modulation as supportive mechanistic evidence only when accompanied by robust metabolic/complication-related endpoints. We highlight the need to account for heterogeneity in preparations, dosing forms, and study quality across preclinical and clinical datasets (including transparent reporting of composition/processing and taxonomically validated source organisms, consistent with GA-online Best Practice/ConPhyMP).

### 2.7 NLRP3 inflammasome: linking inflammatory stress to multi-organ injury

The NLRP3 inflammasome is a vital inflammatory module implicated in T2DM, and its activation promotes caspase-1–dependent maturation of IL-1β and IL-18, thereby contributing to β-cell stress and tissue inflammation. Human- and disease-relevant evidence links NLRP3 activation to T2DM-associated inflammatory outputs, including islet amyloid polypeptide (IAPP)-triggered inflammasome activation and obesity-associated insulin resistance (Masters et al., 2010; Vandanmagsar et al., 2011). Mechanistically, oxidative stress can promote inflammasome activation through the TXNIP–NLRP3 interaction, and NEK7 acts downstream of potassium efflux to enable NLRP3 inflammasome assembly, providing key molecular checkpoints for pathway interpretation (Zhou et al., 2010; He et al., 2016). In this review, we prioritized botanical evidence only when NLRP3-related readouts were accompanied by metabolic or complication-related endpoints. As evidence-linked examples, triptolidiol (a diterpenoid primarily derived from *Tripterygium wilfordii* Hook. f. Celastrol [Celastraceae] inhibits NLRP3 activation by disrupting the NLRP3–NEK7 interaction in cellular and biochemical settings (Ding MY et al., 2025). Triptolide (also primarily associated with *Tripterygium wilfordii* Hook.f. [Celastraceae], as reported) has been reported to ameliorates diabetic nephropathy by inhibiting the NLRP3 inflammasome pathway *in vivo* (Lv et al., 2023). Nevertheless, because inflammasome signaling is pleiotropic, we interpreted NLRP3 modulation as supportive mechanistic evidence only when accompanied by robust outcome-linked readouts (e.g., glycemic indices, tissue injury markers, or complication-related endpoints) and when study quality, controls, and intervention characterization were sufficiently reported (Supplementary Tables S1, S2). Step-specific clarification (revised): In the canonical two-step model, NLRP3 activation involves (i) priming (NF-κB–dependent induction of NLRP3 and pro-IL-1β) and (ii) activation/assembly (NEK7 engagement, ASC oligomerization, caspase-1 cleavage, and IL-1β/IL-18 maturation). Accordingly, we now distinguish that emodin (an anthraquinone commonly associated with *Rheum palmatum L*. [Polygonaceae] and related sources, as reported) is supported mainly as an activation/assembly inhibitor (blocking ASC oligomerization/pro-caspase-1 processing), whereas triptolide is supported mainly as a priming inhibitor via NF-κB. For direct assembly blockade, we cite evidence from the triptolide derivative triptolidiol, which disrupts the NLRP3–NEK7 interaction (Han et al., 2015; Shen et al., 2021; Ding X et al., 2025).

## 3 Cross-organ regulation of the gut–liver–brain–immune axis by TCM in T2DM

**FIGURE 3:**
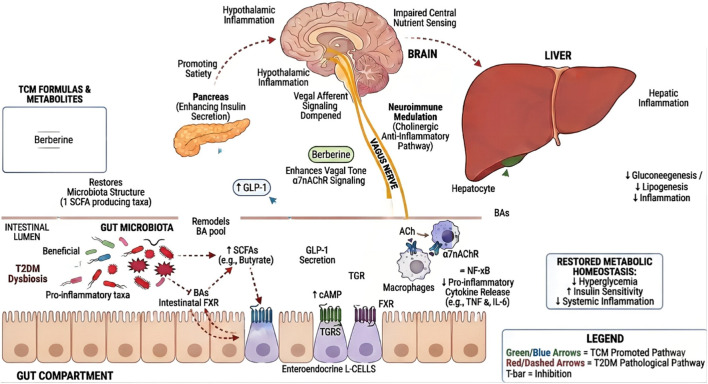
Node-based model of TCM regulation of the gut–liver–brain–immune axis in T2DM. HLJDT and LWDHW (and representative metabolites such as berberine, gardenoside, and rhein) remodel dysbiotic microbiota, increasing SCFA-producing taxa and reshaping the bile acid (BA) pool. SCFAs and BA signaling enhance enteroendocrine GLP-1 release and engage the intestinal FXR/TGR5 pathways, improving insulin secretion and metabolic control. Concurrently, vagus–α7nAChR neuroimmune signaling suppresses NF-κB–linked macrophage cytokine output, whereas hepatic FXR–SHP and CYP7A1-related regulation reduce gluconeogenesis, lipogenesis, and inflammation. Green/blue arrows indicate restorative effects; red/dashed arrows indicate pathological routes; T-bars indicate inhibition.

In recent years, the understanding of T2DM pathogenesis has increasingly moved beyond a purely “insulin metabolic disorder” model toward a framework that emphasizes dysregulated multi-organ crosstalk in systemic metabolic syndrome. In particular, the gut–liver–brain–immune axis is a key cross-organ network governing metabolic homeostasis, neuroendocrine regulation, and inflammatory outputs, and has been implicated in the initiation and progression of T2DM and its multi-system complications. This axis transmits gut microbiota–derived signals to the liver, brain, and immune system through mechanisms that include gut barrier integrity, bile acid signaling, short-chain fatty acids (SCFAs), and vagal-afferent sensing, thereby shaping cross-organ metabolic and immunological regulation. In this context, TCM-related botanical drugs and preparations may offer a complementary, formula-centered framework for modulating multiple nodes of this axis. However, we interpret mechanistic claims cautiously and prioritize evidence supported by outcome-linked readouts and reproducible intervention definitions (including botanical authentication and chemical characterization/QC). Where specific plant metabolites are discussed, we distinguish analytical marker metabolites (used for standardization/QC) from bioactive metabolites (hypothesis-generating candidates requiring orthogonal validation). For the formula-based botanical preparations discussed in this section, complete intervention identity (dosage form, composition, and processing/standardization as reported; NR if not reported) and taxonomically validated source organisms (full scientific names with author citation and family) are provided in Table 2 and Supplementary Table S1. To operationalize this cross-organ axis for formula interpretation, we highlight three relay nodes that connect intestinal ecology to systemic glucose–inflammation phenotypes: (i) bile acid sensing through farnesoid X receptor (FXR) and Takeda G-protein-coupled receptor 5 (TGR5), which tunes enterohepatic metabolism and incretin release; (ii) microbial short-chain fatty acids (SCFAs) engaging GPR41/43 to promote GLP-1 secretion and improve insulin sensitivity; and (iii) neuroimmune signaling via the vagus nerve and α7 nicotinic acetylcholine receptor (α7nAChR) that restrains inflammatory outputs. Using this node-based lens, we further summarized evidence that Huanglian Jiedu Decoction (Huang-Lian-Jie-Du Decoction; HLJDT), a classical multi-herb decoction (identity/QC as reported; NR if not reported; see Table 2/Supplementary Table S1), improves hyperglycemia with concomitant restoration of SCFA-producing microbiota, whereas Liuwei Dihuang Wan (Liu-Wei-Di-Huang Pills; LWDHW), a classical multi-herb pill preparation (identity/QC as reported; NR if not reported; see Table 2/Supplementary Table S1), modulates gut microbiota–SCFA profiles with a proposed SCFAs–GPR43/41–GLP-1 link, thereby providing a tractable mechanistic bridge from classical formulas to cross-organ communication (Katsuma et al., 2005; Trabelsi et al., 2015; Chen et al., 2018; Yi et al., 2022; Tracey, 2002; Wang et al., 2003; Wang D et al., 2022) (Figure 3).

### 3.1 Gut–liver–bile-acid–FXR/TGR5 axis

In addition to lipid absorption, bile acids act as endogenous signaling molecules by activating the farnesoid X receptor (FXR) and G protein-coupled bile acid receptor (TGR5), thereby coordinating glucose–lipid metabolism, inflammatory outputs, and secretion of the gut hormone GLP-1. In T2DM, this axis can be perturbed, with reports describing altered bile acid composition, impaired incretin responses (including reduced GLP-1 output), and amplification of gut–liver inflammatory signaling. Mechanistically, TGR5 activation in enteroendocrine L cells stimulates cAMP signaling and GLP-1 secretion (Katsuma et al., 2005), whereas intestinal FXR activity suppresses proglucagon/GLP-1 production, positioning the BA–FXR/GLP-1 pathway as a bidirectional modulator of glucose homeostasis (Trabelsi et al., 2015). These findings support the view that BA receptor signaling is a “gut–liver–incretin” checkpoint that can be influenced by microbiota-driven bile acid remodeling. In TCM-related research, botanical preparations and representative plant metabolites have been reported to engage multiple nodes within the bile acid receptor network; however, we interpret mechanistic claims cautiously and prioritize studies linking pathway modulation to metabolic or complication-relevant endpoints. For example, the plant metabolite rhein (an anthraquinone primarily associated with *Rheum palmatum* L.[Polygonaceae] and related sources, as reported) has been reported, suppresses CYP7A1 transcription via epigenetic regulation, thereby reducing primary bile acid synthesis and attenuating hepatic inflammatory readouts (Hu et al., 2024). Gardenoside, a plant metabolite (an iridoid glycoside primarily associated with *Gardenia jasminoides* J.Ellis [Rubiaceae]), has been described as a TGR5 agonist associated with increased GLP-1 secretion and improved insulin sensitivity in diabetes-relevant conditions. Berberine, a widely studied plant metabolite (commonly associated with Coptidis Rhizoma; source organisms taxonomically validated and provided in the Supplementary Tables/Supplementary Material, as reported), has been reported to remodel the bile acid pool composition and engage both FXR–small heterodimer partner (SHP) and TGR5–GLP-1 signaling axes (Wang Y. et al., 2024; Xie et al., 2020). Notably, some bile-acid–related metabolites may serve as analytical marker metabolites for standardization/QC rather than proven bioactive metabolites; therefore, causal claims should be supported by orthogonal validation and outcome-linked readouts. Collectively, these studies support the bile acid receptor network as a plausible cross-organ regulatory module relevant to gut–liver axis dysfunction in T2DM.HLJDT has been reported to ameliorate hyperglycemia and insulin resistance in T2DM rats while restoring dysregulated gut microbiota structure and function, notably increasing SCFA-producing taxa and modulating bile acid biosynthesis-related functions (Chen et al., 2018). Consistently, Liuwei Dihuang Wan (Liu-Wei-Di-Huang Pills; LWDHW), a classical multi-herb pill preparation (complete composition and processing/standardization as reported; NR if not reported; see Table 2/Supplementary Table S1), improved glycemic indices and SCFA profiles in GK rats, with mechanistic analyses suggesting the involvement of the SCFAs–GPR43/41–GLP-1 pathway (Yi et al., 2022). Together, these studies illustrate how classical formulas can be interpreted through a microbiota–SCFA–incretin relay, linking intestinal ecology to systemic metabolic control.

### 3.2 Modulating the gut microbiota–SCFA–GLP-1 axis to restore metabolic homeostasis

Beyond metabolic relays, the vagus-mediated cholinergic anti-inflammatory pathway provides a neuroimmune gate in which α7nAChR signaling suppresses pro-inflammatory cytokine release (Tracey, 2002; Pavlov and Tracey, 2005; Wang et al., 2003). In experimental diabetes models, berberine, an isoquinoline alkaloid abundant in *Coptis chinensis Franch. [Ranunculaceae]* and commonly present in HLJDT-related prescriptions, has been shown to improve glucose metabolism via an α7nAChR-dependent mechanism, supporting a plausible vagus–α7nAChR node for formula-related neuroimmune modulation (Wang X et al., 2022). Nevertheless, direct formula-level evidence linking HLJDT/LWDHW to vagal tone or α7nAChR activation remains limited; therefore, we frame this node as hypothesis-generating and requiring targeted validation in formula- and complication-specific settings in the future. As our understanding of the systemic pathogenic mechanisms of T2DM advances, the gut microbiota–SCFA–GLP-1 axis, which links microbiota-derived signals to pancreatic and enteroendocrine function, has emerged as a plausible cross-organ regulatory module relevant to the restoration of metabolic dysregulation. High-fat diets and metabolic dysregulation commonly disrupt the gut microbiota, with reports describing reductions in butyrate-producing taxa (e.g., Faecalibacterium and Roseburia) and decreased SCFA production, particularly butyrate (Zhang et al., 2023). SCFA deficiency is associated with impaired Treg differentiation, compromised gut barrier integrity, and attenuated incretin responses (including TGR5-associated GLP-1 output), thereby worsening insulin resistance and glycemic control (Lin et al., 2025). In the context of TCM-related research, botanical preparations and representative plant metabolites have been reported to engage multiple nodes within this axis; however, we prioritized studies in which microbiota/SCFA changes were accompanied by outcome-linked metabolic readouts (e.g., glycemia and insulin sensitivity indices) and interpreted the associative microbiome findings cautiously. Berberine, a widely studied plant metabolite, has been reported to increase microbiota diversity and enrich butyrate-producing taxa, with concurrent increases in SCFA/butyrate levels and improved metabolic phenotypes, and to modulate gut endocrine signaling, including GLP-1-related pathways (Wang Y et al., 2017; Zhang et al., 2020; Li M. et al., 2020). Gardenoside, a plant metabolite primarily associated with *Gardenia jasminoides J.Ellis [Rubiaceae],* as reported) a plant metabolite described as a TGR5 agonist, has been reported to stimulate GLP-1 secretion and improve insulin-related outcomes (Wang et al., 2022). Rhein, an anthraquinone primarily associated with Rheum palmatum L. [Polygonaceae] and related sources, has been reported to attenuate hepatic inflammation via FXR/SHP-related signaling, supporting improved gut–liver metabolic coupling in diabetes-relevant settings (Zhao et al., 2024). For formula-based botanical preparations mentioned in this section (e.g., HLJDT/LWDHW), complete intervention identity (dosage form, composition, and processing.

Standardization, as reported (NR, if not reported), and taxonomically validated source organisms (full scientific names with author citation and family) are provided in Table 2 and Supplementary Table S1. Notably, some microbiota- and SCFA-associated metabolites may be used as analytical marker metabolites for standardization/QC rather than proven bioactive metabolites; therefore, causal claims should be supported by orthogonal validation and well-controlled experimental designs. These plant metabolites act through a three-stage sequence—“metabolite recognition → immune metabolic remodeling → GLP-1-axis activation”—to restore metabolic homeostasis in the host.

### 3.3 Modulating the gut–brain–vagus nerve axis to enhance central energy sensing

The gut–brain axis integrates neuronal sensing, immune responses, and endocrine signaling to regulate energy intake and systemic metabolic homeostasis. The vagus nerve, a principal conduit linking the gastrointestinal tract to the central nervous system (CNS), plays a key role in conveying gut-derived signals relevant to energy balance (Mayer, 2011). In T2DM, pathological alterations, including impaired gut sensory signaling, hypothalamic inflammation, and POMC neuron dysfunction, are linked to abnormal central nutrient sensing and downstream metabolic dysregulation (Thaler et al., 2012; Caudy et al., 2003). In TCM-related research, multi-botanical preparations and representative plant metabolites have been reported to modulate nodes within gut–brain signaling (e.g., such as vagal-afferent sensing, neuroinflammation, and neuroendocrine outputs). However, we prioritized evidence supported by outcome-linked metabolic readouts and reproducible intervention definitions (including botanical authentication and chemical characterization/quality control). For example, ginsenoside Rg1 (a triterpenoid saponin primarily derived from *Panax ginseng* C.A.Mey. [Araliaceae], as reported) has been reported to modulate cholinergic anti-inflammatory signaling, including its effects on α7 nicotinic acetylcholine receptor (α7nAChR) expression/function in preclinical neuroinflammation-related settings (Feng et al., 2022). In addition, polysaccharides from Wolfiporia extensa (Peck) Ginns [Polyporaceae] (*syn. Poria cocos (*as reported) has been reported to engage innate immune signaling (e.g., TLR2/4–MyD88–NF-κB–related pathways) in preclinical systems, which may indirectly influence gut–brain inflammatory tone, although diabetes-specific causal evidence and gut–vagal endocrine readouts remain limited and should be interpreted cautiously (Chang et al., 2009; Jia et al., 2016). Collectively, these studies support a plausible gut–brain modulatory role for TCM-related interventions, but stronger translational inference requires standardized preparations, appropriate controls, and replication in T2DM-relevant models with clinically meaningful or outcome-linked endpoints (including transparent reporting of source organism identity and extract characterization as reported; NR if not reported, consistent with GA-online Best Practice/ConPhyMP).

## 4 Pharmacological mechanisms of TCM in managing major complications of T2DM

**FIGURE 4:**
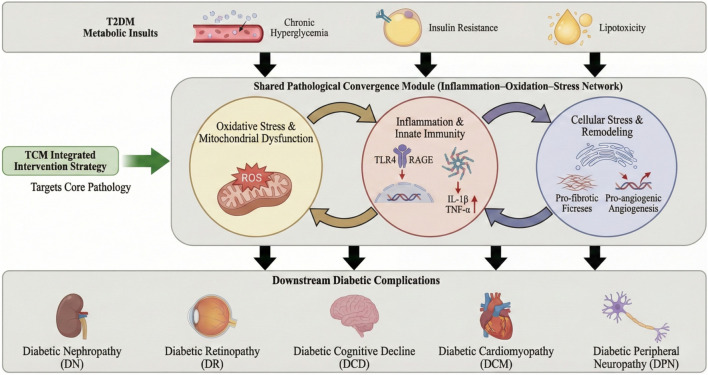
Convergence-module framework linking multi-component TCM to shared drivers and organ-specific complications of T2DM. Upstream metabolic insults (chronic hyperglycemia, insulin resistance, and lipotoxicity) feed into a shared inflammation–oxidation–stress network composed of three clusters: oxidative stress/mitochondrial dysfunction (ROS; NRF2/HO-1 redox defense), innate inflammatory signaling (TLR4/RAGE–NF-κB and NLRP3 inflammasome with IL-1β/TNF-α output), and cellular stress and remodeling (ER stress IRE1α–XBP1; TGF-β/SMADs fibrosis; VEGF angiogenesis). TCM formulas and bioactive metabolites act as integrated, multi-target interventions to restore redox balance and suppress inflammatory cascades, thereby modulating downstream organ-specific pathways in diabetic nephropathy, retinopathy, cognitive decline, cardiomyopathy, and peripheral neuropathy, with corresponding clinical readouts.

T2DM is not merely a disorder of glucose metabolism but a multifaceted metabolic syndrome characterized by multi-system dysregulation (Forbes and Cooper, 2013). The clinical burden of diabetes is largely driven by the onset and progression of multi-organ complications, including diabetic nephropathy (DN), diabetic retinopathy (DR), diabetic cardiomyopathy (DCM), and diabetes-associated cognitive decline (DCD) (Forbes and Cooper, 2013). Although these complications involve diverse tissues and organs, their pathogenic mechanisms substantially overlap, featuring chronic inflammatory activation, impaired redox homeostasis/oxidative stress, compromised insulin signaling, and dysregulated cell death pathways (Forbes and Cooper, 2013; Giacco and Brownlee, 2010). Contemporary standard-of-care therapies (e.g., glucose-lowering agents with cardio–renal benefits) improve glycemic control and reduce complication risk; however, residual risk remains, and disease progression is not fully prevented in many patients, highlighting the need for complementary strategies targeting broader pathogenic modules (American Diabetes Association Professional Practice Committee, 2025a; American Diabetes Association Professional Practice Committee, 2025b). Conventional antidiabetic drugs can also exhibit multi-target and multi-organ actions (e.g.,metformin), reflecting the complexity of T2DM pathophysiology (Rena et al., 2017). In this context, TCM-related multi-botanical preparations (formula-based interventions)—because they comprise multiple constituents—provide a complementary, formula-centered framework that may co-modulate overlapping pathogenic modules (e.g., inflammation, oxidative stress, insulin signaling, and neuroendocrine regulation), thereby supporting integrative organ protection and complication management. Where relevant, we distinguished plant metabolites (e.g., bioactive candidates) from analytical marker metabolites used for standardization/quality control and prioritized evidence in which mechanistic readouts were accompanied by outcome-linked endpoints. This section systematically reviews the research progress and preclinical and clinical evidence on TCM-related botanical preparations for these four representative complications, aiming to offer theoretical support and evidence-based strategies for the comprehensive prevention and treatment of T2DM complications (Figure 4).

### 4.1 Alleviating renal fibrosis and oxidative stress in DN

DN is one of the most common microvascular complications of T2DM and is characterized by thickening of the glomerular basement membrane, mesangial matrix expansion, and interstitial fibrosis (Tervaert et al., 2010). Under persistent hyperglycemia, the TGF-β/SMAD signaling pathway is activated, while advanced glycation end-products (AGEs) and their receptor for advanced glycation end-products (RAGE)-mediated inflammatory cascades are enhanced, accompanied by downregulation of the NRF2 pathway, thereby increasing oxidative stress and aggravating renal injury (Li et al., 2019; Bakris et al., 2010; Guo et al., 2022). Multiple studies have shown that TCM can effectively slow DN progression through multiple target mechanisms. Astragali Radix–related interventions (source species taxonomically validated; e.g., *Astragalus membranaceus* (Fisch.) Bunge [Fabaceae], as reported) downregulates TGF-β expression and activity, thereby reducing collagen deposition and attenuating glomerulosclerosis (Chen and Gould, 2008). Tanshinone IIA (primarily derived from *Salvia miltiorrhiza* Bge. [Lamiaceae], as reported) significantly activates the NRF2/HO-1 pathway, enhancing antioxidant capacity, diminishing reactive oxygen species (ROS) accumulation, and inhibiting cell apoptosis (Wang F et al., 2017). Moreover, Huang Kui capsules (a proprietary botanical preparation; complete composition, dosage form, and processing/standardization/QC as reported; NR if not reported; see Table 2 and Supplementary Table S1) improved the estimated glomerular filtration rate (eGFR) and reduced proteinuria in a multicenter clinical trial, suggesting its potential for renal protection (Gao et al., 2020; Munyangi et al., 2018). Systems pharmacology and network analysis further revealed that TCM formulas in DN intervention can co-regulate core pathways of “inflammation–oxidation–apoptosis,” thereby achieving system-level control of the pathological network. For instance, animal experiments have demonstrated that HLJDT significantly reduces 24-hour urine protein excretion in STZ-induced diabetic rats (∼35% reduction, as reported) and reduces TGF-β1 and collagen I expression in renal tissues (∼40% reduction in immunohistochemistry score, as reported), further supporting its multi-pathway mechanism (Cai et al., 2025).

### 4.2 Suppressing pathological angiogenesis in DR

DR is a predominant cause of adult blindness, with pathogenesis centered on hypoxia-induced upregulation of hypoxia-inducible factor-1α (HIF-1α), which promotes vascular endothelial growth factor (VEGF) expression and triggers aberrant neovascularization (Helmerhorst et al., 2009). Concurrently, hyperglycemia elevates ROS levels and increases vascular permeability, contributing to retinal injury and persistent loss of vision (Hinton et al., 2002). In the context of TCM-related research, botanical preparations and representative plant metabolites have been reported to engage in DR-relevant pathogenic modules (e.g., hypoxia–angiogenesis signaling, oxidative stress, and inflammatory outputs); however, we prioritized studies in which mechanistic readouts were accompanied by outcome-linked retinal endpoints and reproducible intervention definitions. Specifically, ginsenoside Rg1 suppresses HIF-1α expression, thereby attenuating hypoxia-induced angiogenic signaling (Niu et al., 2016). Baicalin inhibits vascular endothelial growth factor receptor 2 (VEGFR2) signaling, with reported reductions in retinal neovascularization in diabetes-relevant settings (Rossino and Casini, 2019). Curcumin and flavonoids from *Cassia obtusifolia L. [Fabaceae]* have been linked to NRF2-related redox responses, mitigating hyperglycemia-associated oxidative stress (Forman and Davies, 2016; Shao et al., 2020). Preclinical evidence further supports these mechanistic observations when paired with disease-relevant outcomes in humans. For example, baicalin has been reported to reduce VEGF expression in high-glucose-induced ARPE-19 retinal epithelial cells and downregulate the TLR4/NF-κB inflammatory pathway (Sabry et al., 2024). In STZ-induced diabetic mice, oral baicalin (50 mg/kg) was associated with a decreased retinal neovascularization area (Gong et al., 2025). At the clinical evidence level, Qiming Granules (a formula-based botanical preparation; complete composition, dosage form, and processing/standardization/QC as reported; NR if not reported; see Table 2 and Supplementary Table S1) in combination with anti-VEGF therapy have been reported to reduce the frequency of vitreous injections and improve macular edema and visual-function–related outcomes, supporting its potential as an adjunctive strategy; nevertheless, these findings should be interpreted in light of trial design, intervention standardization/QC, and outcome definitions (Hu et al., 2021c).

### 4.3 Modulating neuroinflammation and mitochondrial function in diabetes-associated cognitive decline

DCD is a major CNS complication of T2DM that clinically manifests as impaired learning and memory, spatial disorientation, and executive dysfunction (Guimaraes et al., 2023). Studies indicate that DCD pathogenesis reflects convergent mechanisms, including PI3K/Akt dysregulation, mitochondrial dysfunction, and polarization of microglia toward an M1 pro-inflammatory phenotype, which together contribute to neuronal damage and synaptic dysfunction. Meanwhile, sustained activation of TLR4/NF-κB inflammatory signaling is proposed as a disease-relevant inflammatory module in DCD progression, promoting the release of inflammatory mediators (e.g., IL-1β, TNF-α) that can intensify neurotoxicity and cognitive deficits (Mohrin et al., 2021; Lin and Beal, 2006; Miah et al., 2020; Peters et al., 2021). With the proposal of the “neuro-inflammation–metabolism coupling” network concept, regulating key nodes within this network has gradually become a potential intervention for diabetes-related brain dysfunction. In this context, TCM-related research has garnered interest because of its reported ability to modulate neuroimmune–metabolic interactions. Multiple studies suggest that TCM-related botanical preparations and representative plant metabolites may confer system-level protection in DCD via multi-target mechanisms, although we prioritized evidence in which mechanistic readouts are accompanied by outcome-linked neurobehavioral or neuropathological endpoints and reproducible intervention definitions. For example, *Ginkgo biloba L.* [Ginkgoaceae] extract (standardization/characterization as reported; NR if not reported) has been reported to engage the PI3K/Akt/BDNF axis, with associated improvements in synaptic plasticity and neurorepair-related outcomes (Diaz-Amarilla et al., 2022). Gastrodin (a phenolic glycoside primarily associated with *Gastrodia elata* Blume [Orchidaceae]) has been reported to inhibit TLR4/NF-κB signaling, with concurrent reductions in CNS inflammatory markers (Wong et al., 2023). Liuwei Dihuang Pill has been reported to upregulate hippocampal BDNF levels and improve learning and memory in T2DM animal models (Behl et al., 2022). Subsequent experimental studies have further supported the neuroprotective potential of TCM-related interventions in cognitive impairment models. In the APP/PS1 transgenic mouse model, ginsenoside Rg1 has been reported to improve spatial memory performance, with concurrent changes in hippocampal BDNF protein expression and Akt phosphorylation, suggesting that its cognitive effects may involve PI3K/Akt–BDNF-linked signaling in that model context (Zhang et al., 2025; Nie et al., 2017). For formula-based botanical preparations and extracts discussed in this section, intervention identity (composition and processing/standardization as reported; NR if not reported) and taxonomically validated source organisms (full scientific names including author citation and family) are provided in Table 2 and Supplementary Table S1.

### 4.4 Enhancing antioxidant defense and reducing myocardial stress in diabetic cardiomyopathy

DCM is a common and severe cardiovascular complication in T2DM patients, characterized by myocardial hypertrophy, impaired diastolic function, and a substantially increased risk of heart failure (Badran et al., 2024). Unlike vascular heart diseases such as atherosclerosis, DCM primarily manifests as intrinsic myocardial structural and functional abnormalities that are not solely explained by epicardial coronary artery disease. This pathological progression is associated with mitochondrial dysfunction, sustained endoplasmic reticulum stress (ERS), and disrupted calcium homeostasis (Bugger and Abel, 2014; Jia et al., 2018). At the molecular level, convergent stress responses involving redox dysregulation and ERS signaling have been proposed as key drivers of cardiomyocyte injury and functional decline in DCM patients. Notably, downregulation of the NRF2–antioxidant response element (ARE) pathway compromises the endogenous antioxidant capacity of the cardiomyocytes (Barancik et al., 2016). In parallel, abnormal activation of the IRE1α–XBP1 axis may amplify cellular stress and pro-apoptotic signaling, thereby exacerbating myocardial damage and decompensation. In TCM-related research, botanical preparations and representative plant metabolites have been reported to engage stress response modules in DCM-relevant models. For example, icariin (a prenylated flavonol glycoside primarily associated with *Epimedium brevicornu* Maxim. [Berberidaceae] and related Epimedium sources, as reported) has been reported to activates NRF2-related signaling, increases SOD2-associated antioxidant readouts, reduces lipid peroxidation (MDA-related readouts), and suppresses the IRE1α–XBP1 axis, consistent with a reduced ERS burden (Li C et al., 2022; Zheng Y. et al., 2022; Zheng J. et al., 2022). Network pharmacology analyses further suggested that TCM-related interventions may co-modulate multiple nodes across the AMPK, PI3K/Akt, Ca2+/calmodulin-dependent protein kinase II (CaMKII), and related signaling pathways. However, we interpret *in silico* pathway–target networks as hypothesis-generating unless supported by outcome-linked experimental validation. For the formula-based botanical preparations discussed in this section, intervention identity (dosage form, complete composition, and processing/standardization as reported; NR if not reported) and taxonomically validated source organisms (full scientific names including author citation and family) are provided in Table 2 and Supplementary Table S1. At the metabolite level, tanshinone IIA, a representative plant metabolite from the TCM-related botanical drug Salvia, has been reported to exhibit antioxidant and antiapoptotic properties in cell-based and animal models. In high-glucose-treated H9c2 cardiomyocytes, the reported IC_50_ is 12.6 μM (as reported), with concurrent increases in SOD2 expression and reductions in malondialdehyde (MDA) content (Ji et al., 2018). In STZ-induced diabetic mice, tanshinone IIA treatment was associated with reduced cardiac TUNEL-positive cells, suggesting potential myocardial protection in that model, possibly involving Nrf2/HO-1–linked responses (Moura et al., 2021). Nevertheless, mechanistic reports remain heterogeneous in terms of model choice, exposure/dose ranges, and intervention definition/standardization (including botanical authentication and chemical characterization/QC), and pathway interactions are not consistently tested using orthogonal readouts. Therefore, further studies should prioritize reproducible intervention definitions, dose–response characterization with appropriate controls and outcome-linked endpoints to clarify the key regulatory targets and translational potential of anti-DCM strategies.

### 4.5 Addressing diabetic peripheral neuropathy (DPN): transient receptor potential vanilloid 1 (TRPV1)-Linked nociceptive sensitization and PI3K/Akt-mediated neurorepair

Diabetic peripheral neuropathy (DPN) is one of the most prevalent and disabling complications of T2DM and commonly presents as distal symmetric neuropathy with pain, paresthesia, and sensory loss, substantially impairing the quality of life and increasing the risk of foot ulceration and amputation (Pop-Busui et al., 2017; Eid et al., 2023). Given its high disease burden and close association with metabolic stress and microvascular dysfunction, we added DPN as a dedicated complication section to align the chapter structure with clinical epidemiology and therapeutic needs. Mechanistically, DPN reflects the convergence of hyperglycemia- and dyslipidemia-driven bioenergetic failure, axon–Schwann cell dysfunction, and neuroimmune activation. In painful DPN, peripheral sensitization is critically shaped by the remodeling of nociceptors and ion channels. TRPV1 in dorsal root ganglion neurons is a well-supported effector of diabetic neuropathic pain and can be potentiated by upstream inflammatory signaling (Xie et al., 2022; Wang A et al., 2021; Chen et al., 2022). Accordingly, an integrative anti-DPN strategy should combine the suppression of neuroinflammatory amplification with the restoration of neurorepair programs (e.g., Schwann-cell autophagy/myelination and axonal support), rather than focusing on analgesia alone (Eid et al., 2023; Yang et al., 2025). From the TCM perspective, “blood-activating” and “Qi-tonifying” herbs frequently used in T2DM-related neuropathic symptom clusters—such as Angelica sinensis (Oliv.) Diels [Apiaceae], *Ligusticum chuanxiong* Hort. [Apiaceae], and *Astragalus membranaceus* (Fisch.) Bunge [Fabaceae]can be interpreted in modern neurobiology as modulators of pain-related ion channels and pro-repair signaling. A TRP-channel-oriented literature synthesis indicates that multiple TCM herbs/ingredients regulate thermo-TRP channels (including TRPV1) relevant to nociceptive sensitization (Yan et al., 2022). Importantly, astragaloside IV—a triterpenoid saponin primarily associated with *Astragalus membranaceus* (Fisch.) Bunge [Fabaceae], as reported, attenuates Schwann cell injury and improves peripheral nerve function in DPN models, in part through miR-155–linked autophagy regulation, with involvement of the upstream PI3K/Akt/mTOR axis (Yin et al., 2021). In addition, Danggui-containing classical prescriptions, such as Danggui Sini Decoction (Danggui-Sini Decoction; a classical multi-herb decoction; complete composition, dosage form, and processing/standardization/QC as reported; NR if not reported; see Table 2 and Supplementary Table S1), have shown experimental/clinical evidence of alleviating diabetic neuropathic pain with reduced glial activation and pro-inflammatory cytokines in the spinal cord, providing a tractable bridge between traditional practice and neuroinflammation-focused mechanisms (Liu et al., 2017).

### 4.6 Integrated TCM strategies for multi-organ complication management in T2DM: common mechanisms and graph-based therapeutic modeling

#### 4.6.1 Shared pathophysiological mechanisms among T2DM complications

Although DN, DR, DCM, and DCD manifest in distinct organs, they share a hierarchical pathobiological cascade rather than a single linear axis. Upstream metabolic insults (chronic hyperglycemia, insulin resistance, and lipotoxicity) activate canonical biochemical pathways (AGE–RAGE signaling, polyol/hexosamine flux, and PKC activation) and establish metabolic/epigenetic “memory,” which drives endothelial dysfunction and microvascular injury. These vascular changes intersect with mitochondrial dysfunction/redox imbalance, ER stress, impaired autophagy/mitophagy, and inflammatory signaling (including TLR4–NF-κB and NLRP3 inflammasome activation with downstream IL-1β/IL-18 and pyroptotic cell death). Downstream, each organ expresses dominant remodeling outputs: transforming growth factor beta (TGF-β)/SMAD-mediated fibrosis in the kidney and heart (DN/DCM), vascular endothelial growth factor (VEGF)-driven pathological angiogenesis and barrier breakdown in the retina (DR), and neurovascular unit dysfunction with mixed vascular–neurodegenerative pathology in the brain (DCD). Therefore, we revise the “oxidative stress–inflammation/NLRP3” triad from a unifying label to a central convergence module embedded within a broader network that includes vascular dysfunction, metabolic memory, and organ-specific remodeling programs, providing a more accurate and comprehensive integrative framework for multi-organ complication management in T2DM (Brownlee, 2005; Forbes and Cooper, 2013; Chen and Natarajan, 2022; Xue et al., 2023; Li et al., 2023; Biessels and Despa, 2018).

#### 4.6.2 Multi-target synergy of TCM in multi-organ regulation

TCM emphasizes a strategy of “syndrome differentiation–based treatment, multi-pathway integration, and holistic regulation,” conferring multi-target and multi-system synergy for system-level disease intervention. Research indicates that certain TCM formulas can target multiple pathological aspects of T2DM in a single intervention. Botanical preparations containing Astragali Radix (source species taxonomically validated; e.g., *Astragalus membranaceus* (Fisch.) Bunge [Fabaceae], as reported) and *Salvia miltiorrhiza* Bge. [Lamiaceae] have been reported to engage PI3K/Akt- and NRF2–HO-1–related signaling, consistent with the mitigation of oxidative-stress–linked injury in cardiovascular and nervous-system–relevant models. Furthermore, *Panax ginseng* C. A. Meyer [Araliaceae] and *Scutellaria baicalensis* Georgi [Lamiaceae]-related interventions have been reported to modulate TLR4/NF-κB and HIF-1α/VEGF signaling, consistent with the suppression of pathological angiogenesis in retinal and neurovascular contexts. The clinical evidence was interpreted in an evidence-graded manner and is summarized in Supplementary Table S2. For ocular outcomes in diabetic retinopathy, randomized trial syntheses of oral Chinese commercial Chinese polyherbal preparation (CCPP) support improvements in visual and retinal endpoints, although the primary study reporting quality is frequently limited (Liu et al., 2023). For diabetic nephropathy–related renal outcomes, meta-analytic evidence suggests the potential benefits of Liuwei Dihuang–based interventions (formula identity, composition, and processing/standardization as reported; NR if not reported; see Table 2 and Supplementary Table S1) on renal function/proteinuria, but heterogeneity and trial-quality limitations remain important (Gao et al., 2018). Accordingly, we avoid over-interpreting pathway “hits” from network pharmacology or single-assay readouts, given assay interference/PAINS liabilities, and prioritize studies with outcome-linked endpoints and reproducible intervention definitions (Baell and Holloway, 2010; Dahlin et al., 2021).

#### 4.6.3 Building a multi-dimensionall graph-based TCM intervention map for T2DM

It is essential to establish a Five-Dimensional Intervention Map (FDIM) centered on the core dimensions of “botanical preparation (formula)–plant metabolite–pathway–organ–disease” to enable systematic modeling and visual representation of TCM interventions for T2DM complications. This map, which integrates system pharmacology, multi-omics, and graph neural networks (GNNs), comprehensively describes the cross-level regulatory pathways of multi-botanical formulations. By embedding “botanical preparation–plant metabolite–target–pathway–organ–disease–phenotype” relationships in a heterogeneous GNN, intervention pathway inference, plant metabolite contribution ranking, and key target identification can be achieved in the context of comorbid diseases (Lin et al., 2020; Shi et al., 2022). Graph attention networks (GATs) may further improve the identification of core action nodes (e.g., PI3K, NLRP3, and BDNF) and enhance the biological interpretability of the model (Wolf et al., 2011; Paparozzi et al., 2025). Moreover, the integration of spatial transcriptomics with organoid platforms enables tissue-level validation of map-predicted targets and pathways, thereby closing the loop from mechanistic prediction to experimental verification and advancing the systematic modernization of TCM pharmacological research (Wesemann, 2022; Ho et al., 2020). In conclusion, the potential value of TCM-related interventions for T2DM multi-organ complications may stem from integrated module-level co-regulation. Building a multi-dimensional graph map model could help uncover action networks of multi-botanical preparations and provide a structured decision-support framework for personalized intervention strategies, while distinguishing analytical marker metabolites (for QC) from bioactive candidate metabolites (for efficacy hypotheses).

## 5 Clinical evidence and modernization challenges of TCM in T2DM

**FIGURE 5:**
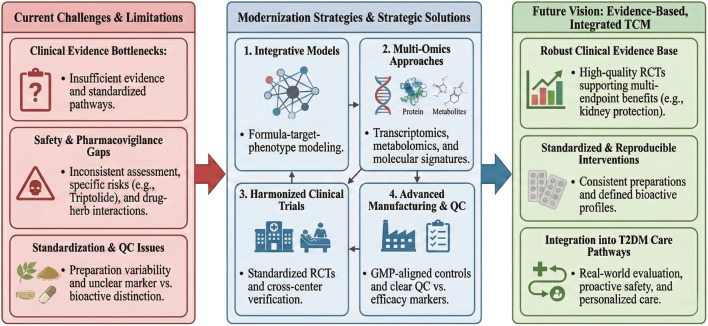
Roadmap for modernizing evidence-based integrated TCM in T2DM care. The schematic summarizes the current bottlenecks in clinical evidence/evaluation, safety and pharmacovigilance (including herb–drug interactions and compound-specific risks), and standardization/quality control of botanical preparations. Proposed solutions include integrative system models (formula–metabolite–target–phenotype mapping), multi-omics profiling to quantify exposure-linked signatures, harmonized multicenter clinical trials with standardized endpoints, and GMP-aligned manufacturing with third-party quality control and traceability. Together, these steps support a future vision of robust clinical evidence, standardized and reproducible interventions, and pragmatic integration of TCM into T2DM care pathways with proactive safety monitoring and personalized systems-level management.

Although TCM-related botanical preparations (multi-botanical preparations) have accumulated substantial experimental and theoretical evidence for engaging multiple T2DM-relevant pathogenic modules, their clinical translation within contemporary healthcare systems is constrained by several limitations. Key bottlenecks include an insufficiently evidence-graded clinical evaluation framework, delayed or inconsistent safety assessments, and a lack of standardized and internationally aligned pathways for implementation and dissemination. In addition, translation is often hindered by variability in botanical drug sourcing and the standardization of botanical preparations, including incomplete quality control strategies that distinguish analytical marker metabolites (for QC/standardization) from bioactive candidate metabolites (for efficacy). In recent years, advances in evidence-based medicine have yielded an increasing number of higher-quality randomized controlled trials (RCTs) evaluating TCM-related botanical preparations for T2DM, which helps strengthen the clinical evidence base for modernization and supports more standardized clinical application (Figure 5).

### 5.1 Accumulated evidence from randomized controlled trials

Numerous randomized controlled trials (RCTs) and meta-analyses have reported that TCM-related multi-botanical preparations (formula-based interventions) can improve key metabolic endpoints in T2DM, supporting an evidence base that is strongest when the intervention is clearly defined and standardized. HLJDT combined with metformin has been reported to decrease HbA1c levels by approximately 1.3% (as reported) and improve (reduce) insulin resistance (replace “(18)” with a standard author–year citation). Regarding complications, Qiming Granules (a formula-based botanical preparation; intervention identity and QC/standardization as reported; NR if not reported; see Table 2 and Supplementary Table S1) combined with anti-VEGF therapy have been reported to reduce intravitreal injection frequency and alleviate macular edema (Zhang et al., 2024). In renal protection, a multicenter RCT study involving 268 T2DM patients demonstrated that Huang Kui Capsules (a proprietary botanical preparation; intervention identity and QC/standardization as reported; NR if not reported; see Table 2 and Supplementary Table S1) stabilized the estimated glomerular filtration rate (eGFR) and reduced proteinuria levels (Wen et al., 2025). Across studies, interpretation should consider concomitant therapies, dosage forms, and quality control, including the use of analytical marker metabolites for standardization (distinct from hypothesized bioactive candidate metabolites). Collectively, these findings suggest that TCM-related botanical preparations may provide multi-endpoint, multi-organ benefits in glycemic control, organ protection, and complication management, and motivate further well-designed trials and pragmatic/real-world evaluations to support their integration into contemporary T2DM care.

### 5.2 Pharmacovigilance and safety profiling of TCM interventions

Although most TCM formulas are derived from classic pharmacopoeias and are widely used in practice, potential safety risks remain in contemporary clinical settings, especially in long-term interventions and multi-drug combinations. Certain bioactive plant metabolites, such as triptolide and rhein, may induce transaminase elevation or nephrotoxicity at high doses (Hu et al., 2022; Cheng et al., 2020). Several studies have indicated that TCM-related botanical drugs (e.g., Angelica sinensis (Oliv.) Diels [Apiaceae], *Salvia miltiorrhiza* Bge. [Lamiaceae], Glycyrrhiza uralensis Fisch. ex DC. [Fabaceae] (and/or other Glycyrrhiza source species, as reported)) may markedly increase the risk of bleeding when used in conjunction with anticoagulants or antiplatelet medications (e.g., warfarin and aspirin) (Li LR et al., 2022). Although the Chinese Pharmacopoeia institutes certain quality control criteria for TCM, systematic construction of adverse event databases, dose–toxicity thresholds, and evidence of chronic toxicology remain insufficient. Therefore, it is urgent to build a multi-dimensional safety assessment framework focused on “plant metabolite identification (including analytical marker metabolites for QC)–metabolic characteristics–toxicity mechanisms–pharmacokinetics,” combining real-world evidence and pharmacoepidemiology platforms to achieve dynamic monitoring and precise management of TCM clinical safety. Notably, the diterpenoid epoxide triptolide (primarily derived from Tripterygium wilfordii Hook. f. [Celastraceae], as reported) represents a well-recognized high-risk TCM-derived compound with a narrow therapeutic window. Experimental and clinical reports have linked it to dose- and time-dependent hepatotoxicity (e.g., ALT/AST elevation and cholestatic features) and nephrotoxicity. Although certain Tripterygium preparations (intervention identity and processing/standardization as reported; NR if not reported) provide approximate clinical dosing ranges (e.g., daily triptolide exposure in the hundreds of micrograms), a universally accepted human “toxicity threshold” is not clearly defined and is further modified by CYP/P-gp–mediated exposure, co-medications, and host susceptibility. For anthraquinones, emodin (present in Rheum spp.; source species taxonomically validated as reported; see Supplementary Table S1) has been reported to show bidirectional hepatic effects with toxicity signals at high concentrations/prolonged exposure and kidney injury signals in some toxicology datasets. However, dose–toxicity boundaries remain inconsistent across models, and sub-chronic animal studies at moderate doses may show minimal hepatic/renal injury. Therefore, in long-term administration and polypharmacy settings, we emphasize a pragmatic safety principle: prioritize standardized preparations, avoid concomitant hepatotoxic/nephrotoxic drugs when Possible, and implement periodic liver and renal function monitoring (ALT/AST, bilirubin, creatinine/eGFR), especially when the formulas contain Tripterygium-derived components or anthraquinone-rich herbs (Hu et al., 2022; Xi et al., 2017; Dong et al., 2016; Sougiannis et al., 2021).

### 5.3 Standardization challenges and strategies for modern integration

TCM emphasizes “syndrome differentiation–based treatment,” which may offer advantages for personalized care but also poses substantial challenges for modern drug evaluation in terms of the standardization and reproducibility of results. These challenges include variability between botanical preparations (e.g., decoctions, granules, and pills), inconsistent sources of raw botanical drugs, diverse processing methods, large batch-to-batch fluctuations in key plant metabolites, and a lack of systematic comparisons of bioavailability and pharmacokinetic (PK) profiles across preparations. To promote the modernization of TCM evaluation systems, four areas should be prioritized: (i) building a “formula–plant metabolite–target–phenotype” integrative model and establishing mechanistic fingerprinting to improve interpretability and consistency; (ii) applying multi-omics and high-throughput platforms (e.g.,transcriptomics, metabolomics, proteomics) to quantify exposure-linked molecular response signatures and activity features of botanical preparations; (iii) developing harmonized RCT design standards and enabling cross-center verification through national-level clinical research platforms; and (iv) advancing GMP-aligned manufacturing and supply chain controls, including third-party quality control and traceability systems, to achieve controlled consistency in formula quality, with an explicit distinction between analytical marker metabolites (for QC) and bioactive metabolites (as candidate effectors).

## 6 Integrating cutting-edge technologies into TCM research for T2DM: from mechanistic discovery to predictive modeling

With rapid advances in systems biology, artificial intelligence (AI), spatial multi-omics, and graph neural networks (GNNs), research on TCM-related botanical drugs and multi-botanical preparations is increasingly shifting from an experience-driven paradigm to an integrated “mechanistic discovery–model prediction” workflow. In complex metabolic diseases such as T2DM, these technologies can enhance the systems-level interpretation of how botanical preparations and their plant metabolites relate to disease-relevant modules, while also enabling data-driven optimization of formula composition, prioritization of candidate targets, and hypothesis generation for personalized strategies. Importantly, such modeling frameworks should explicitly distinguish analytical marker metabolites (used for standardization/quality control) from bioactive metabolites (candidate effectors) to improve reproducibility and translational interpretability. The following sections summarize how these emerging approaches can support mechanistic discoveries and model-driven predictions in TCM-related research.

### 6.1 AI for mechanism elucidation and compound-formula prioritization in TCM

AI, particularly natural language processing (NLP) and graph-based learning algorithms, is gradually becoming a core tool for knowledge mining and mechanism modeling in TCM research (Duan et al., 2024; Long et al., 2025). NLP enables the automatic extraction of entities such as “botanical drug–syndrome–target–signaling pathway–disease” and their interrelationships from classical TCM literature, structured databases, and modern clinical research. This facilitates the construction of TCM knowledge graphs (KGs) to achieve cross-hierarchical mechanism inference and multi-botanical preparation intervention pathway prediction (Weng et al., 2022; Zhuang et al., 2025). Graph neural networks (GNNs), built on KGs, can model complex heterogeneous relationships among “formula-based botanical preparations–plant metabolites–targets–pathways–phenotypes,” integrating topological structures and attribute features to identify key plant metabolites and generate hypotheses on synergistic mechanisms. For example, in studies of Gegen Qinlian Decoction (Gegen-Qinlian Decoction; GQD; a classical multi-herb decoction; complete composition and processing/standardization/QC as reported; NR if not reported; see Table 2 and Supplementary Table S1) in T2DM, GNN-based analyses have been used to prioritize key signaling pathways, such as PI3K/Akt, NF-κB, and AMPK, and to map putative high-contribution plant metabolites and pathway-level action networks (Xu et al., 2021). Further integration of classical machine-learning models, such as Random Forest (RF) and XGBoost, can enable the efficient screening of candidate bioactive metabolites and target prediction scoring, thereby proposing an efficient methodology for the accelerated development and more precise evaluation of candidate multi-botanical preparations (with experimental and outcome-linked validation required for translational inference).

### 6.2 Multi-omics integration for mechanism-compound formulation-phenotype closed-loop modeling in TCM

The systemic metabolic dysregulation characteristic of T2DM, combined with the multi-target nature of multi-botanical preparation interventions, underscores the limitations of single-omics approaches in holistically elucidating their mechanisms of action. Integrated multi-omics strategies, including metabolomics, transcriptomics (particularly single-cell RNA sequencing (scRNA-seq)), proteomics, and spatial transcriptomics, have emerged as pivotal methodologies for deciphering the pharmacological mechanisms underlying TCM-related botanical preparations. Metabolomics enables the comprehensive profiling of TCM-related botanical preparation–associated remodeling of glucose, lipid, and bile acid metabolic networks (Shao et al., 2022). Transcriptomics reveals pathway-specific alterations and cell subtype-specific responses (Wang et al., 2020), whereas spatial transcriptomics further delineates the spatial localization of formula-level actions at the tissue microenvironment level (Qian et al., 2024). For instance, a multi-omics study on Huanglian Jiedu Decoction (Huanglian-Jiedu Decoction; HLJDT) suggests that its intervention mechanism involves the “gut–liver–brain–adipose” multi-axis network, with putative engagement of key nodes such as AMPK, FXR, and NLRP3, and supports a multi-level workflow that encompasses mechanism identification, tissue localization, and phenotypic validation (Dai et al., 2024). These strategies can support systematic pharmacodynamic modeling and outcome-linked mechanism validation for multi-botanical preparations, provided that intervention definition/standardization and appropriate controls are transparently reported, thereby advancing the interpretability of TCM-related research.

### 6.3 Advancing TCM mechanistic research: novel biological models for innovation and validation

To enhance the biological explanatory power of mechanistic modeling, an increasing number of studies on TCM-related botanical preparations have incorporated advanced functional validation platforms such as organoids, organ-on-a-chip (OoC) systems, and spatial multi-omics. These models more faithfully recapitulate the structural and functional complexity of *in vivo* tissue microenvironments, enabling dynamic and tissue-specific analyses of multi-botanical preparations (Zhao et al., 2022; Low et al., 2021; Larsson et al., 2021). For instance, evidence suggests that ginsenoside Rg1, a representative plant metabolite frequently investigated in the TCM research context (primarily associated with *Panax ginseng* C. A. Mey. [Araliaceae], as reported), may improve central energy homeostasis and ameliorate cognitive impairment in T2DM models by activating POMC neurons and the PI3K/Akt–BDNF pathway, a mechanism amenable to precise spatial and functional validation using hypothalamic organoid platforms (Feng et al., 2022; Jiang et al., 2023). This integrated “mechanism–botanical preparation–tissue” experimental framework may help strengthen standardization, reproducibility, and spatially resolved mechanistic validation in TCM-related research.

### 6.4 Deciphering multi-target mechanisms of TCM with GNNs

GNNs are emerging as pivotal technical tools in system pharmacology research on TCM, owing to their capacity to model complex, heterogeneous relational structures and their growing adoption in molecular/drug-discovery graph learning (Abate et al., 2023). By constructing multi-layered graphs encompassing “botanical preparation–plant metabolite–target–pathway–organ–disease” relationships, GNNs can be used to support multiple core functions: (1) intervention path inference, predicting potential multi-pathway regulatory trajectories of multi-botanical preparations within comorbidity networks; (2) node importance ranking, identifying key plant metabolites or targets with central regulatory roles; and (3) synergistic target mapping, identifying shared targets across pathways to elucidate convergent mechanisms and network-level integration. For instance, a study utilized a GNN-based pharmacology graph model to predict that Yuquan Pill (a formula-based botanical preparation; complete composition, dosage form, and processing/standardization/QC as reported; NR if not reported; see Table 2 and Supplementary Table S1) may synergistically improve cardiac and renal functions via the AMPK–ACC–NRF2 signaling axis, suggesting its potential mechanism in the intervention of multiple organ complications in T2DM (Jiang et al., 2023). Looking ahead, coupling graph learning with spatially resolved multi-omics can improve biological interpretability by anchoring predictions to tissue microenvironments and cell-type-specific contexts (Marx, 2021). In summary, emerging technologies such as AI, multi-omics, spatial biology, and graph learning are profoundly reshaping the paradigm of TCM research, providing hypothesis-generating and decision-support capabilities for the systematic analysis and mechanism modeling of TCM-related botanical preparations (with translational inference requiring standardized interventions, appropriate controls, and outcome-linked validation).

## 7 Conclusion and future perspectives

Type 2 diabetes mellitus (T2DM) is a complex systemic metabolic syndrome characterized by multi-system dysregulation involving interconnected pathological mechanisms, such as insulin resistance, chronic inflammation, oxidative stress, and neuro-immune dysregulation. It is frequently accompanied by multi-organ complications involving the heart, kidneys, brain, and eyes. TCM-related botanical drugs and preparations are commonly delivered as multi-constituent, formula-based interventions that can engage multiple biological targets and pathways in the body. In this review, we use this feature primarily as a systems-level framework to organize evidence across shared pathogenic modules in T2DM rather than implying that “multi-target” pharmacology is unique to TCM formulations. This review systematically synthesizes the key mechanisms through which TCM-related interventions may ameliorate T2DM, including modulation of signaling pathways, such as PI3K/Akt and AMPK, inflammatory outputs (NF-κB/NLRP3), remodeling of the gut–liver–brain–immune network, and pathways relevant to diabetic complications. Where available, we prioritized evidence in which pathway modulation was accompanied by metabolic or complication-relevant endpoints (e.g., HbA1c, insulin sensitivity indices, and tissue injury markers) and interpreted pathway-only findings with caution. Given this substantial epidemiological burden, the real-world impact of TCM on diabetes management should be interpreted through an evidence-based lens. Beyond mechanistic studies, future work should prioritize well-designed randomized trials, pragmatic studies, and real-world evaluations with transparent reporting of botanical preparation standardization, dosage forms, concomitant therapies, safety outcomes, and clinically meaningful endpoints (e.g., HbA1c, complication progression, and patient-reported outcomes). In line with GA-online best-practice recommendations, intervention identity is treated as a prerequisite for interpretation: for each botanical preparation discussed, composition and processing/standardization (including quality control/chemical characterization) are recorded as reported, and items not provided in the primary study are explicitly marked as NR (not reported), rather than being inferred. All botanical source species mentioned in this review are taxonomically validated and reported with full scientific names (including authorities) and families, with details provided in Table 2 and in Supplementary Table S1.

Assay interference and PAINS considerations are particularly important when interpreting the effects of plant metabolites. Some molecules may appear active across multiple biochemical or cell-based assays because of non-target-specific mechanisms (e.g., redox cycling, fluorescence/absorbance interference, or colloidal aggregation) rather than genuine target engagement. To strengthen translational confidence, future studies should incorporate orthogonal assay formats and counter-screens (e.g., detergent sensitivity for aggregation, alternative readouts, and appropriate negative controls) before concluding the target-specific pharmacology of plant metabolites. Taken together, these assay-level considerations highlight the broader limitations of the current evidence base and motivate clear research priorities for future translation. In line with the individualized practice emphasized by TCM, an emerging direction is to operationalize constitution typing (e.g., Wang Qi’s nine-constitution framework) as a stratification layer for the integrated risk prediction and management of T2DM. Standardized questionnaires, such as the Constitution in Chinese Medicine Questionnaire (CCMQ), enable scalable phenotyping, and multiple studies have linked unbalanced constitutions (e.g., phlegm-dampness, damp-heat, and qi deficiency) to inflammatory/adipokine profiles and a higher risk of diabetes or adverse outcomes. Therefore, future “TCM + systems” models could incorporate constitution types as interpretable nodes alongside clinical variables, multi-omics signatures, and medication exposure to support constitution-informed prevention, complication surveillance, and personalized formula selection (Wong et al., 2013; You et al., 2017; Bai YL et al., 2021; Lee et al., 2024). Limitations and research requirements First, the clinical evidence is heterogeneous and frequently limited by suboptimal trial design and reporting (e.g., incomplete randomization/blinding details, short follow-up, and variable endpoints), underscoring the need to follow established guidelines for herbal interventions and systematic reviews (Gagnier et al., 2006; Page et al., 2021). Second, many mechanistic findings rely on simplified *in vitro*/*in silico* models or non-physiological exposures, with insufficient dose–response characterization and unclear minimal active concentrations/doses, which constrain model-to-human extrapolations. Third, reproducibility is weakened by inadequate intervention standardization and extract characterization (e.g., missing marker quantification, batch consistency, and chemical profiling). Future studies should align with the best practice recommendations for phytopharmacological reporting and characterization (Heinrich et al., 2022). Fourth, rigorous controls and orthogonal validation are inconsistently applied, and phytochemical “target” claims should be interpreted cautiously, given assay interference/PAINS liabilities, and other non-specific effects (Baell and Holloway, 2010; Dahlin et al., 2021). Finally, to close the translation gap, more pragmatic trials and high-quality real-world evaluations with transparent standardization and clinically meaningful endpoints are needed, complemented by target trial emulation principles when RCTs are not feasible (Ford and Norrie, 2016; Hernán and Robins, 2016).

Furthermore, emerging technologies, such as GNNs, multi-omics integration, spatial transcriptomics, and organoid modeling, are progressively enabling the construction of a five-dimensional interventional map linking “formula–plant metabolite–pathway–organ–disease.” This structured framework supports the transition of TCM from an experience-based system to a mechanism-driven research platform. Based on these limitations, we outline the following translational priorities and future research directions: Looking ahead, the systematic integration of TCM in T2DM therapy holds promise through advances in five key areas: ① closed-loop validation of “mechanism–plant metabolite–phenotype” links to establish causal evidence chains; ② spatially resolved target engagement and pathway verification enabled by spatial multi-omics, organoids, and organ-on-a-chip platforms; ③ GNN-enhanced systems-pharmacology modeling to improve the interpretability of multi-botanical preparation effects and to prioritize testable hypotheses; ④ development of dynamic safety databases and internationally aligned standardization/quality-control frameworks (including botanical authentication, marker-based QC, batch consistency, and dosage-form transparency) to support reproducibility and clinical reliability; and ⑤ AI-assisted knowledge mining to guide indication expansion and rational, personalized combination strategies, with explicit attention to drug–botanical interaction risk and real-world implementability. In summary, TCM research is evolving toward a modern pharmacology platform characterized by mechanism-oriented, evidence-graded analyses, reproducible intervention definitions, and clinically meaningful translational endpoints. Its potential role in managing complex chronic diseases, such as T2DM, should be judged based on outcome-linked evidence and reproducible reporting standards, with translation strengthened by rigorous trials, pragmatic designs, and real-world evaluations.

## References

[B177] Abate C. Decherchi S. Cavalli A. (2023). Graph neural networks for conditional de novo drug design. WIREs Comput. Mol. Sci. 13, e1651. 10.1002/wcms.1651

[B1] American Diabetes Association Professional Practice Committee (2025a). Cardiovascular disease and risk management: standards of care in Diabetes—2025. Diabetes Care 48 (Suppl. 1), S207–S238. 10.2337/dc25-S010 39651970 PMC11635050

[B2] American Diabetes Association Professional Practice Committee (2025b). Chronic kidney disease and risk management: standards of care in Diabetes—2025. Diabetes Care 48 (Suppl. 1), S239–S251. 10.2337/dc25-S011 39651975 PMC11635029

[B3] An Y. Xu B.-T. Wan S.-R. Ma X.-M. Long Y. Xu Y. (2023). The role of oxidative stress in diabetes mellitus-induced vascular endothelial dysfunction. Cardiovasc Diabetol. 22, 237. 10.1186/s12933-023-01965-7 37660030 PMC10475205

[B4] Badran H. M. Helmy J. A. Ahmed N. F. Yacoub M. (2024). Impact of diabetes mellitus on left ventricular mechanics and long-term outcome in patients with hypertrophic cardiomyopathy. Echocardiography 41 (12), e70048. 10.1111/echo.70048 39661017

[B5] Baell J. B. Holloway G. A. (2010). New substructure filters for removal of pan assay interference compounds (PAINS) from screening libraries and for their exclusion in bioassays. J. Med. Chem. 53, 2719–2740. 10.1021/jm901137j 20131845

[B6] Bai F. Luo H. Wang L. Zhu L. Guan Y. Zheng Y. (2021). A meta-analysis of the association between diabetes mellitus and traditional Chinese medicine constitution. Evid. Based Complement. Altern. Med. 2021, 6390530. 10.1155/2021/6390530 34394389 PMC8357480

[B183] Bai Y. Mu Q. Bao X. Zuo J. Fang X. Hua J. (2021). Targeting NLRP3 inflammasome in the treatment of diabetes and diabetic complications: role of natural compounds from herbal medicine. Aging Dis. 12 (7), 1587–1604. 10.14336/AD.2021.0318 34631209 PMC8460305

[B7] Bai Y. L. Han L. L. Qian J. H. Wang H. Z. (2021). Molecular mechanism of puerarin against diabetes and its complications. Front. Pharmacol. 12, 780419. 10.3389/fphar.2021.780419 35058775 PMC8764238

[B8] Bakris G. Vassalotti J. Ritz E. Wanner C. Stergiou G. Molitch M. (2010). National kidney foundation consensus conference on cardiovascular and kidney diseases and diabetes risk: an integrated therapeutic approach to reduce events. Kidney Int. 78 (8), 726–736. 10.1038/ki.2010.292 20720529

[B9] Barancik M. Gresova L. Bartekova M. Dovinova I. (2016). Nrf2 as a key player of redox regulation in cardiovascular diseases. Physiol. Res. 65 (Suppl. 1), S1–S10. 10.33549/physiolres.933403 27643930

[B10] Behl T. Kaur I. Sehgal A. Singh S. Sharma N. Makeen H. A. (2022). Aducanumab making a comeback in Alzheimer's disease: Aa old wine in a new bottle. Biomed. Pharmacother. 148, 112746. 10.1016/j.biopha.2022.112746 35231697

[B11] Biessels G. J. Despa F. (2018). Cognitive decline and dementia in diabetes mellitus: mechanisms and clinical implications. Nat. Rev. Endocrinol. 14, 591–604. 10.1038/s41574-018-0048-7 30022099 PMC6397437

[B12] Brownlee M. (2005). The pathobiology of diabetic complications. Diabetes 54 (6), 1615–1625. 10.2337/diabetes.54.6.1615 15919781

[B13] Bugger H. Abel E. D. (2014). Molecular mechanisms of diabetic cardiomyopathy. Diabetologia 57 (4), 660–671. 10.1007/s00125-014-3171-6 24477973 PMC3969857

[B14] Butler A. E. Janson J. Bonner-Weir S. Ritzel R. Rizza R. A. Butler P. C. (2003). Beta-cell deficit and increased beta-cell apoptosis in humans with type 2 diabetes. Diabetes 52 (1), 102–110. 10.2337/diabetes.52.1.102 12502499

[B15] Cai R. Li C. Zhao Y. Yuan H. Zhang X. Liang A. (2025). Traditional Chinese medicine in diabetic kidney disease: multifaceted therapeutic mechanisms and research progress. Chin. Med. 20 (1), 95. 10.1186/s13020-025-01150-w 40598250 PMC12211247

[B16] Caudy A. A. Ketting R. F. Hammond S. M. Denli A. M. Bathoorn A. M. Tops B. B. (2003). A micrococcal nuclease homologue in RNAi effector complexes. Nature 425 (6956), 411–414. 10.1038/nature01956 14508492

[B179] Chang H. H. Yeh C. H. Sheu F. (2009). A novel immunomodulatory protein from Poria cocos induces toll-like receptor 4-dependent activation within mouse peritoneal macrophages. J. Agric. Food Chem. 57 (14), 6129–6139. 10.1021/jf9011399 19548679

[B17] Chen T. M. Gould M. (2008). Evaluation of patients with small, subcentimeter nodules. Semin. Respir. Crit. Care Med. 29 (3), 241–247. 10.1055/s-2008-1076744 18506662

[B18] Chen Z. Natarajan R. (2022). Epigenetic modifications in metabolic memory: what are the memories, and can we erase them? Am. J. Physiol. Cell Physiol. 323 (2), C570–C582. 10.1152/ajpcell.00201.2022 35785987 PMC9359656

[B19] Chen M. Liao Z. Lu B. Wang M. Lin L. Zhang S. (2018). Huang-Lian-Jie-Du-Decoction ameliorates hyperglycemia and insulin resistant in association with Gut microbiota modulation. Front. Microbiol. 9, 2380. 10.3389/fmicb.2018.02380 30349514 PMC6186778

[B20] Chen W. Yuan C. Lu Y. Zhu Q. Ma X. Xiao W. (2020). Tanshinone IIA protects against acute pancreatitis in mice by inhibiting oxidative stress via the Nrf2/ROS pathway. Oxidative Med. Cell. Longev. 2020, 5390482. 10.1155/2020/5390482 32322336 PMC7168729

[B21] Chen L. Wang H. Xing J. Shi X. Huang H. Huang J. (2022). Silencing P2X7R alleviates diabetic neuropathic pain involving TRPV1 via PKCε/P38MAPK/NF-κB signaling pathway in rats. Int. J. Mol. Sci. 23 (22), 14141. 10.3390/ijms232214141 36430617 PMC9696864

[B22] Cheng C.-W. Wu T.-X. Shang H.-C. Li Y.-P. Altman D. G. Moher D. (2017). CONSORT extension for Chinese herbal medicine formulas 2017: recommendations, explanation, and elaboration. Ann. Intern. Med. 167 (2), 112–121. 10.7326/M16-2977 28654980

[B23] Cheng Y. Zhang H. Qu L. He Y. Routledge M. N. Yun G. Y. (2020). Identification of rhein as the metabolite responsible for toxicity of rhubarb anthraquinones. Food Chem. 331, 127363. 10.1016/j.foodchem.2020.127363 32590269

[B24] Dahlin J. L. Auld D. S. Rothenaigner I. Haney S. Sexton J. Z. Nissink J. W. M. (2021). Nuisance compounds in cellular assays. Cell Chem. Biol. 28, 356–370. 10.1016/j.chembiol.2021.01.021 33592188 PMC7979533

[B25] Dai J. Qiu L. Lu Y. Li M. (2024). Recent advances of traditional Chinese medicine against cardiovascular disease: overview and potential mechanisms. Front. Endocrinol. 15, 1366285. 10.3389/fendo.2024.1366285 39403576 PMC11471557

[B26] Defronzo R. A. (2009). Banting lecture. From the triumvirate to the ominous octet: a new paradigm for the treatment of type 2 diabetes mellitus. Diabetes 58 (4), 773–795. 10.2337/db09-9028 19336687 PMC2661582

[B27] Diaz-Amarilla P. Arredondo F. Dapueto R. Boix V. Carvalho D. Santi M. D. (2022). Isolation and characterization of neurotoxic astrocytes derived from adult triple transgenic Alzheimer's disease mice. Neurochem. Int. 159, 105403. 10.1016/j.neuint.2022.105403 35853553

[B28] Ding M.-Y. Ning C. Chen S.-R. Yin H.-R. Xu J. Wang Y. (2025). Discovery of natural product derivative triptolidiol as a direct NLRP3 inhibitor by reducing K63-specific ubiquitination. Br. J. Pharmacol. 182 (20), 4876–4893. 10.1111/bph.17320 39219027

[B29] Ding X. Lv M. Yu Y. Chen Y. Zhou X. Huang X. (2025). Triptolidiol blocks NLRP3 inflammasome activation by preventing NLRP3–NEK7 interaction. Br. J. Pharmacol. 182, 4876–4893. 10.1111/bph.17135 39219027

[B30] Dong X. Fu J. Yin X. Cao S. Li X. Lin L. (2016). Emodin: a review of its pharmacology, toxicity and pharmacokinetics. Phytother. Res. 30 (8), 1207–1218. 10.1002/ptr.5631 27188216 PMC7168079

[B31] Du X. Matsumura T. Edelstein D. Rossetti L. Zsengeller Z. Szabo C. (2003). Inhibition of GAPDH activity by poly(ADP-ribose) polymerase activates three major pathways of hyperglycemic damage in endothelial cells. J. Clin. Invest 112 (7), 1049–1057. 10.1172/JCI18127 14523042 PMC198524

[B32] Duan Y. Zhou Q. Li Y. Qin C. Wang Z. Kan H. (2024). Research on a traditional Chinese medicine case-based question-answering system integrating large language models and knowledge graphs. Front. Med. 11, 1512329. 10.3389/fmed.2024.1512329 39839612 PMC11747316

[B33] Eid S. A. Rumora A. E. Beirowski B. Bennett D. L. Hur J. Savelieff M. G. (2023). New perspectives in diabetic neuropathy. Neuron 111 (17), 2623–2641. 10.1016/j.neuron.2023.05.003 37263266 PMC10525009

[B34] Feng H. Xue M. Deng H. Cheng S. Hu Y. Zhou C. (2022). Ginsenoside and its therapeutic potential for cognitive impairment. Biomolecules 12 (9), 1310. 10.3390/biom12091310 36139149 PMC9496100

[B35] Forbes J. M. Cooper M. E. (2013). Mechanisms of diabetic complications. Physiol. Rev. 93 (1), 137–188. 10.1152/physrev.00045.2011 23303908

[B36] Ford I. Norrie J. (2016). Pragmatic trials. N. Engl. J. Med. 375 (5), 454–463. 10.1056/NEJMra1510059 27518663

[B37] Forman H. J. Davies K. (2016). Commentary on “Bach1 differentially regulates distinct Nrf2-dependent genes in human venous and coronary artery endothelial cells adapted to physiological oxygen levels” by Chapple et al. Free. Radic. Biol. Med. 92, 163–164. 10.1016/j.freeradbiomed.2016.01.013 26802904

[B38] Fu Y. J. Xu B. Huang S. W. Luo X. Deng X. L. Luo S. (2021). Baicalin prevents LPS-induced activation of TLR4/NF-kappaB p65 pathway and inflammation in mice via inhibiting the expression of CD14. Acta Pharmacol. Sin. 42 (1), 88–96. 10.1038/s41401-020-0411-9 32457419 PMC7921675

[B39] GA-online (2025). Best practice in research—ConPhyMP. Available online at: https://ga-online.org/best-practice/ (Accessed December 16, 2025).

[B40] Gagnier J. J. Boon H. Rochon P. Moher D. Barnes J. Bombardier C. (2006). Reporting randomized, controlled trials of herbal interventions: an elaborated CONSORT statement. Ann. Intern Med. 144 (5), 364–367. 10.7326/0003-4819-144-5-200603070-00013 16520478

[B174] Gao H. Duan Y. Fu X. Xie H. Liu Y. Yuan H. (2018). Comparison of efficacy of SHENQI compound and rosiglitazone in the treatment of diabetic vasculopathy analyzing multi-factor mediated disease-causing modules. PLoS One 13 (12), e0207683. 10.1371/journal.pone.0207683 30521536 PMC6283585

[B41] Gao L. Liu J. Xu P. Deng G. Liu B. Yuan F. (2020). AKT inhibitor SC66 inhibits proliferation and induces apoptosis in human glioblastoma through down-regulating AKT/beta-Catenin pathway. Front. Pharmacol. 11, 1102. 10.3389/fphar.2020.01102 32848734 PMC7411127

[B42] Gao W. Y. Chen P. Y. Hsu H. J. Lin C. Y. Wu M. J. Yen J. H. (2021). Tanshinone IIA downregulates lipogenic gene expression and attenuates lipid accumulation by modulation LXRalpha/SREBP1 pathway in HepG2 cells. Biomedicines 9 (3), 326. 10.3390/biomedicines9030326 33806955 PMC8004631

[B43] GBD 2021 Diabetes Collaborators (2023). Global, regional, and national burden of diabetes from 1990 to 2021, with projections of prevalence to 2050: a systematic analysis for the global Burden of disease study 2021. Lancet 402 (10397), 203–234. 10.1016/S0140-6736(23)01301-6 37356446 PMC10364581

[B44] Ghafouri-Fard S. Balaei N. Shoorei H. Hasan S. Hussen B. M. Talebi S. F. (2022). The effects of ginsenosides on PI3K/AKT signaling pathway. Mol. Biol. Rep. 49 (7), 6701–6716. 10.1007/s11033-022-07270-y 35220526 PMC9270311

[B45] Ghareghomi S. Rahban M. Moosavi-Movahedi Z. Habibi-Rezaei M. Saso L. Moosavi-Movahedi A. A. (2021). The potential role of curcumin in modulating the master antioxidant pathway in diabetic hypoxia-induced complications. Molecules 26 (24), 7658. 10.3390/molecules26247658 34946740 PMC8706440

[B46] Giacco F. Brownlee M. (2010). Oxidative stress and diabetic complications. Circ. Res. 107 (9), 1058–1070. 10.1161/CIRCRESAHA.110.223545 21030723 PMC2996922

[B47] Goldfine A. B. Shoelson S. E. (2017). Therapeutic approaches targeting inflammation for diabetes and associated cardiovascular risk. J. Clin. Invest. 127, 83–93. 10.1172/JCI88884 28045401 PMC5199685

[B48] Goldfine A. B. Fonseca V. Jablonski K. A. Pyle L. Staten M. A. Shoelson S. E. (2010). The effects of salsalate on glycemic control in patients with type 2 diabetes: a randomized trial. Ann. Intern. Med. 152, 346–357. 10.7326/0003-4819-152-6-201003160-00004 20231565 PMC3138470

[B49] Goldfine A. B. Conlin P. R. Halperin F. Koska J. Permana P. Schwenke D. (2013). A randomised trial of salsalate for insulin resistance and cardiovascular risk factors in persons with abnormal glucose tolerance. Diabetologia 56, 714–723. 10.1007/s00125-012-2819-3 23370525 PMC4948114

[B50] Gong P. Xiao X. Wang S. Shi F. Liu N. Chen X. (2023). Corrigendum to: “hypoglycemic effect of astragaloside IV *via* modulating gut microbiota and regulating AMPK/SIRT1 and PI3K/AKT pathway” [J. Ethnopharmacol. 281 (2021) 114558]. J. Ethnopharmacol. 313, 116629. 10.1016/j.jep.2023.116629 37188618

[B51] Gong T. Wang D. Wang J. Huang Q. Zhang H. Liu C. (2025). Study on the mechanism of plant metabolites to intervene oxidative stress in diabetic retinopathy. Front. Pharmacol. 16, 1517964. 10.3389/fphar.2025.1517964 39974734 PMC11835683

[B52] Guimaraes I. Costa R. Madureira S. Borges S. Oliveira A. L. Pintado M. (2023). Tannic acid tailored-made microsystems for wound infection. Int. J. Mol. Sci. 24 (5), 4826. 10.3390/ijms24054826 36902255 PMC10003198

[B53] Guo J. Jie W. Shen Z. Li M. Lan Y. Kong Y. (2022). [Corrigendum] SCF increases cardiac stem cell migration through PI3K/AKT and MMP-2/-9 signaling. Int. J. Mol. Med. 49 (3), 36. 10.3892/ijmm.2022.5091 35088875 PMC8815413

[B54] Han J. W. Shim D. W. Shin W. Y. Heo K. H. Kwak S. B. Sim E. J. (2015). Anti-inflammatory effect of emodin via attenuation of NLRP3 inflammasome activation. Int. J. Mol. Sci. 16 (4), 8102–8109. 10.3390/ijms16048102 25867480 PMC4425069

[B55] He Y. Zeng M. Y. Yang D. Motro B. Núñez G. (2016). Necroptosis promotes NLRP3 inflammasome activation and IL-1β production in macrophages. Nature 530 (7591), 354–357. 10.1038/nature16934 26814970 PMC4810788

[B56] Heinrich M. Jalil B. Abdel-Tawab M. Echeverria J. Kulić Ž. McGaw L. J. (2022). Best practice in the chemical characterisation of extracts used in pharmacological and toxicological research—the ConPhyMP—Guidelines. Front. Pharmacol. 13, 953205. 10.3389/fphar.2022.953205 36176427 PMC9514875

[B57] Helmerhorst H. J. Wijndaele K. Brage S. Wareham N. J. Ekelund U. (2009). Objectively measured sedentary time may predict insulin resistance independent of moderate- and vigorous-intensity physical activity. Diabetes 58 (8), 1776–1779. 10.2337/db08-1773 19470610 PMC2712788

[B58] Hernán M. A. Robins J. M. (2016). Using big data to emulate a target trial when a randomized trial is not available. Am. J. Epidemiol. 183 (8), 758–764. 10.1093/aje/kww098 26994063 PMC4832051

[B59] Herzig S. Shaw R. J. (2018). AMPK: guardian of metabolism and mitochondrial homeostasis. Nat. Rev. Mol. Cell Biol. 19 (2), 121–135. 10.1038/nrm.2017.95 28974774 PMC5780224

[B60] Hinton D. R. He S. Jin M. L. Barron E. Ryan S. J. (2002). Novel growth factors involved in the pathogenesis of proliferative vitreoretinopathy. EYE 16 (4), 422–428. 10.1038/sj.eye.6700190 12101449

[B61] Ho S. H. Zhang C. Tao F. Zhang C. Chen W. H. (2020). Microalgal torrefaction for solid biofuel production. Trends. Biotechnol. 38 (9), 1023–1033. 10.1016/j.tibtech.2020.02.009 32818442

[B62] Hopkins A. L. (2008). Network pharmacology: the next paradigm in drug discovery. Nat. Chem. Biol. 4 (11), 682–690. 10.1038/nchembio.118 18936753

[B63] Hu Z. Liu X. Yang M. (2021a). Evidence and potential mechanisms of Jin-Gui Shen-Qi wan as a treatment for type 2 diabetes mellitus: a systematic review and meta-analysis. Front. Pharmacol. 12, 699932. 10.3389/fphar.2021.699932 34552482 PMC8450514

[B64] Hu Z. Xie C. Yang M. Fu X. Gao H. Liu Y. (2021b). Add-on effect of Qiming granule, a Chinese patent medicine, in treating diabetic macular edema: a systematic review and meta-analysis. Phytother. Res. 35 (2), 587–602. 10.1002/ptr.6844 32939932

[B65] Hu Z. Yang M. Liu Y. Yang Q. Xie H. Peng S. (2021c). Effect of Huang-Lian Jie-Du decoction on glucose and lipid metabolism in type 2 diabetes mellitus: a systematic review and meta-analysis. Front. Pharmacol. 12, 648861. 10.3389/fphar.2021.648861 33995064 PMC8117159

[B66] Hu Y. Wu Q. Wang Y. Zhang H. Liu X. Zhou H. (2022). The molecular pathogenesis of triptolide-induced hepatotoxicity. Front. Pharmacol. 13, 979307. 10.3389/fphar.2022.979307 36091841 PMC9449346

[B67] Hu Z. Cheng X. Cai J. Huang C. Hu J. Liu J. (2024). Emodin alleviates cholestatic liver injury by modulating Sirt1/Fxr signaling pathways. Sci. Rep. 14 (1), 16756. 10.1038/s41598-024-67882-1 39033253 PMC11271454

[B68] Huang F. Liu K. Du H. Kou J. Liu B. (2012). Puerarin attenuates endothelial insulin resistance through inhibition of inflammatory response in an IKKbeta/IRS-1-dependent manner. Biochimie 94 (5), 1143–1150. 10.1016/j.biochi.2012.01.018 22314193

[B69] Huang X. Liu G. Guo J. Su Z. (2018). The PI3K/AKT pathway in obesity and type 2 diabetes. Int. J. Biol. Sci. 14 (11), 1483–1496. 10.7150/ijbs.27173 30263000 PMC6158718

[B185] International Diabetes Federation (2025). IDF Diabetes Atlas. 11th Edn. Brussels, Belgium: International Diabetes Federation.

[B70] Ji P. Fan X. Ma X. Wang X. Zhang J. Mao Z. (2018). Kruppel-like factor 9 suppressed tumorigenicity of the pancreatic ductal adenocarcinoma by negatively regulating frizzled-5. Biochem. Biophys. Res. Commun. 499 (4), 815–821. 10.1016/j.bbrc.2018.03.229 29621541

[B180] Jia G. DeMarco V. G. Sowers J. R. (2016). Insulin resistance and hyperinsulinaemia in diabetic cardiomyopathy. Nat. Rev. Endocrinol. 12 (3), 144–153. 10.1038/nrendo.2015.216 26678809 PMC4753054

[B71] Jia G. Whaley-Connell A. Sowers J. R. (2018). Diabetic cardiomyopathy: a hyperglycaemia- and insulin-resistance-induced heart disease. Diabetologia 61 (1), 21–28. 10.1007/s00125-017-4390-4 28776083 PMC5720913

[B72] Jiang N. Lv J. Zhang Y. Sun X. Yao C. Wang Q. (2023). Protective effects of ginsenosides Rg1 and Rb1 against cognitive impairment induced by simulated microgravity in rats. Front. Pharmacol. 14, 1167398. 10.3389/fphar.2023.1167398 37168997 PMC10164943

[B73] Jorgensen S. B. O'Neill H. M. Sylow L. Honeyman J. Hewitt K. A. Palanivel R. (2013). Deletion of skeletal muscle SOCS3 prevents insulin resistance in obesity. Diabetes 62 (1), 56–64. 10.2337/db12-0443 22961088 PMC3526029

[B74] Katsuma S. Hirasawa A. Tsujimoto G. (2005). Bile acids promote glucagon-like peptide-1 secretion through TGR5 in a murine enteroendocrine cell line STC-1. Biochem. Biophys. Res. Commun. 329 (1), 386–390. 10.1016/j.bbrc.2005.01.139 15721318

[B75] Kearney A. L. Norris D. M. Ghomlaghi M. Kin L. W. M. Humphrey S. J. Carroll L. (2021). Akt phosphorylates insulin receptor substrate to limit PI3K-mediated PIP3 synthesis. Elife 10, e66942. 10.7554/eLife.66942 34253290 PMC8277355

[B182] Larsson L. Frisén J. Lundeberg J. (2021). Spatially resolved transcriptomics adds a new dimension to genomics. Nat. Methods 18 (1), 15–18. 10.1038/s41592-020-01038-7 33408402

[B76] Lee Y. S. Kim W. S. Kim K. H. Yoon M. J. Cho H. J. Shen Y. (2006). Berberine, a natural plant product, activates AMP-activated protein kinase with beneficial metabolic effects in diabetic and insulin-resistant states. Diabetes 55 (8), 2256–2264. 10.2337/db06-0006 16873688

[B77] Lee C.-H. Su Y.-C. Lin S.-Y. Lee I.-T. Tsai C.-I. Li T.-C. (2024). Associations of traditional Chinese medicine body constitution and all-cause mortality in patients with type 2 diabetes mellitus: a prospective cohort study of a Taiwanese medical center. Front. Med. 10, 1320861. 10.3389/fmed.2023.1320861 38249989 PMC10797087

[B184] Lei Y. Huang J. Xie Z. Wang C. Li Y. Hua Y. (2023). Elucidating the pharmacodynamic mechanisms of Yuquan pill in T2DM rats through comprehensive multi-omics analyses. Front. Pharmacol. 14, 1282077. 10.3389/fphar.2023.1282077 38044947 PMC10691276

[B78] Li C. Jia W. W. Yang J. L. Cheng C. Olaleye O. E. (2022). Multi-compound and drug-combination pharmacokinetic research on Chinese herbal medicines. Acta Pharmacol. Sin. 43 (12), 3080–3095. 10.1038/s41401-022-00983-7 36114271 PMC9483253

[B79] Li L. Zhong S. Shen X. Li Q. Xu W. Tao Y. (2019). Recent development on liquid chromatography-mass spectrometry analysis of oxidized lipids. Free. Radic. Biol. Med. 144, 16–34. 10.1016/j.freeradbiomed.2019.06.006 31202785

[B80] Li Y. Teng D. Shi X. Qin G. Qin Y. Quan H. (2020). Prevalence of diabetes recorded in mainland China using 2018 diagnostic criteria from the American diabetes association: national cross sectional study. BMJ 369, m997. 10.1136/bmj.m997 32345662 PMC7186854

[B81] Li Y. Liu Y. Liu S. Gao M. Wang W. Chen K. (2023). Diabetic vascular diseases: molecular mechanisms and therapeutic strategies. Signal Transduct. Target Ther. 8, 152. 10.1038/s41392-023-01400-z 37037849 PMC10086073

[B171] Li Y-P. Altman D. G. Moher D. (2017). CONSORT Extension for Chinese Herbal Medicine Formulas 2017: Recommendations, Explanation, and Elaboration. Ann. Intern. Med. 167 (2), 112–121. 10.7326/M16-2977 28654980

[B82] Li L. R. Sethi G. Zhang X. Liu C. L. Huang Y. Liu Q. (2022). The neuroprotective effects of icariin on ageing, various neurological, neuropsychiatric disorders, and brain injury induced by radiation exposure. Aging (Albany NY) 14 (3), 1562–1588. 10.18632/aging.203893 35165207 PMC8876913

[B83] Li M. Zhou W. Dang Y. Li C. Ji G. Zhang L. (2020). Berberine compounds improves hyperglycemia *via* microbiome mediated colonic TGR5-GLP pathway in db/db mice. Biomed. Pharmacother. 132, 110953. 10.1016/j.biopha.2020.110953 33254441

[B84] Lin M. T. Beal M. F. (2006). Mitochondrial dysfunction and oxidative stress in neurodegenerative diseases. Nature 443 (7113), 787–795. 10.1038/nature05292 17051205

[B85] Lin X. Quan Z. Wang Z. J. Huang H. Zeng X. (2020). A novel molecular representation with BiGRU neural networks for learning atom. Brief. Bioinform. 21 (6), 2099–2111. 10.1093/bib/bbz125 31729524

[B86] Lin M. Zhang H. Liu S. Li A. Nan Z. (2023). Efficacy of “Dihuang pill prescriptions” combined with conventional treatment for diabetic kidney disease: a network meta-analysis and systematic review. Med. Baltim. 102 (39), e35290. 10.1097/MD.0000000000035290 37773831 PMC10545219

[B87] Lin X. Xia L. Zhou Y. Xie J. Tuo Q. Lin L. (2025). Crosstalk between bile acids and intestinal epithelium: multi-Dimensional roles of farnesoid X receptor and takeda G protein receptor 5. Int. J. Mol. Sci. 26 (9), 4240. 10.3390/ijms26094240 40362481 PMC12072030

[B88] Liu M. Qiu H. Q. Qian L. Chang X. Y. Li X. Liu S. (2017). Effects of Danggui Sini decoction on neuropathic pain: experimental studies and clinical pharmacological significance of inhibiting glial activation and pro-inflammatory cytokines in the spinal cord. Int. J. Clin. Pharmacol. Ther. 55 (5), 453–464. 10.5414/CP202613 28372633

[B173] Liu S. Li A. Nan Z. (2023). Efficacy of “Dihuang pill prescriptions” combined with conventional treatment for diabetic kidney disease: network meta-analysis and systematic review. Medicine (Baltimore) 102 (3), e35290. 10.1097/MD.0000000000035290 37773831 PMC10545219

[B89] Long J. Niu J. Wang H. Liu J. Li J. Zhang W. (2025). RTGN: robust traditional Chinese medicine graph networks for patient similarity learning. IEEE J. Biomed. Health Inf. 29 (3), 2185–2198. 10.1109/JBHI.2024.3510884 40030577

[B176] Low L. A. Mummery C. Berridge B. R. Austin C. P. Tagle D. A. (2021). Organs-on-chips: into the next decade. Nat. Rev. Drug Discov. 20 (5), 345–361. 10.1038/s41573-020-0079-3 32913334

[B90] Luan X. Zhang L.-J. Li X.-Q. Rahman K. Zhang H. Chen H.-Z. (2020). Compound-based Chinese medicine formula: from discovery to compatibility mechanism. J. Ethnopharmacol. 254, 112687. 10.1016/j.jep.2020.112687 32105748

[B91] Luo X.-x. Duan J.-g. Liao P.-z. Wu L. Yu Y.-g. Qiu B. (2009). Effect of qiming granule on retinal blood circulation of diabetic retinopathy: a multicenter clinical trial. Chin. J. Integr. Med. 15 (5), 384–388. 10.1007/s11655-009-0384-5 19802544

[B92] Lv C. Cheng T. Zhang B. Sun K. Lu K. (2023). Triptolide protects against podocyte injury in diabetic nephropathy by activating the Nrf2/HO-1 pathway and inhibiting the NLRP3 inflammasome pathway. Ren. Fail. 45, 2165103. 10.1080/0886022X.2023.2165103 36938748 PMC10035962

[B178] Marx V. (2021). Method of the Year: spatially resolved transcriptomics. Nat. Methods 18 (1), 9–14. 10.1038/s41592-020-01033-y 33408395

[B93] Masters S. L. Dunne A. Subramanian S. L. Hull R. L. Tannahill G. M. Sharp F. A. (2010). Activation of the NLRP3 inflammasome by islet amyloid polypeptide provides a mechanism for enhanced IL-1β in type 2 diabetes. Nat. Immunol. 11, 897–904. 10.1038/ni.1935 20835230 PMC3103663

[B94] Mayer E. A. (2011). Gut feelings: the emerging biology of gut-brain communication. Nat. Rev. Neurosci. 12 (8), 453–466. 10.1038/nrn3071 21750565 PMC3845678

[B95] Miah M. R. Ijomone O. M. Okoh C. Ijomone O. K. Akingbade G. T. Ke T. (2020). The effects of manganese overexposure on brain health. Neurochem. Int. 135, 104688. 10.1016/j.neuint.2020.104688 31972215 PMC7926190

[B96] Mohrin M. Liu J. Zavala-Solorio J. Bhargava S. Maxwell T. J. Brito A. (2021). Inhibition of longevity regulator PAPP-A modulates tissue homeostasis via restraint of mesenchymal stromal cells. Aging Cell 20 (3), e13313. 10.1111/acel.13313 33561324 PMC7963332

[B97] Moura D. de Sales I. Brandao J. A. Costa M. R. Queiroz C. M. (2021). Disentangling chemical and electrical effects of status epilepticus-induced dentate gyrus abnormalities. Epilepsy Behav. 121 (Pt B), 106575. 10.1016/j.yebeh.2019.106575 31704249

[B98] Munyangi J. Cornet-Vernet L. Idumbo M. Lu C. Lutgen P. Perronne C. (2018). Effect of Artemisia annua and Artemisia afra tea infusions on schistosomiasis in a large clinical trial. Phytomedicine 51, 233–240. 10.1016/j.phymed.2018.10.014 30466622 PMC6990975

[B99] Ng C. Zhong L. Ng H. S. Goh K. S. Zhao Y. (2024). Managing type 2 diabetes mellitus via the regulation of Gut microbiota: a Chinese medicine perspective. Nutrients 16 (22), 3935. 10.3390/nu16223935 39599721 PMC11597546

[B100] Nie L. Xia J. Li H. Zhang Z. Yang Y. Huang X. (2017). Ginsenoside Rg1 ameliorates behavioral abnormalities and modulates the hippocampal proteomic change in triple transgenic mice of Alzheimer's disease. Oxidative Med. Cell. Longev. 2017, 6473506. 10.1155/2017/6473506 29204248 PMC5674513

[B101] Niu B. Liu L. Su H. Xia X. He Q. Feng Y. (2016). Role of extracellular signal-regulated kinase 1/2 signal transduction pathway in insulin secretion by beta-TC6 cells. Mol. Med. Rep. 13 (5), 4451–4454. 10.3892/mmr.2016.5053 27035884

[B102] Page M. J. McKenzie J. E. Bossuyt P. M. Boutron I. Hoffmann T. C. Mulrow C. D. (2021). The PRISMA 2020 statement: an updated guideline for reporting systematic reviews. BMJ 372, n71. 10.1136/bmj.n71 33782057 PMC8005924

[B103] Paparozzi V. Hooshmandabbasi R. Ravoni A. Ma Y. Manni L. Koh T. J. (2025). Anti-inflammatory effects of physical stimuli: the central role of networks in shaping the future of pharmacological research. Br. J. Pharmacol., bph.70129. 10.1111/bph.70129 40702933

[B104] Pavlov V. A. Tracey K. J. (2005). The cholinergic anti-inflammatory pathway. Brain Behav. Immun. 19 (6), 493–499. 10.1016/j.bbi.2005.03.015 15922555

[B105] Peng S. Xie Z. Zhang X. Xie C. Kang J. Yuan H. (2021). Efficacy and safety of the Chinese patent medicine Yuquan pill on type 2 diabetes mellitus patients: a systematic review and meta-analysis. Evid. Based Complement. Altern. Med. 2021, 2562590. 10.1155/2021/2562590 34899945 PMC8660199

[B106] Peters V. T. A. Leijte G. P. Franssen G. M. Bruse N. Booij J. Doorduin J. (2021). Human *in vivo* neuroimaging to detect reprogramming of the cerebral immune response following repeated systemic inflammation. Brain. Behav. Immun. 95, 321–329. 10.1016/j.bbi.2021.04.004 33839233

[B107] Pop-Busui R. Boulton A. J. M. Feldman E. L. Bril V. Freeman R. Malik R. A. (2017). Diabetic neuropathy: a position statement by the American diabetes association. Diabetes Care 40 (1), 136–154. 10.2337/dc16-2042 27999003 PMC6977405

[B108] Qian Y. Xiong S. Li L. Sun Z. Zhang L. Yuan W. (2024). Spatial multi-omics atlas reveals smooth muscle phenotypic transformation and metabolic reprogramming in diabetic macroangiopathy. Cardiovasc. Diabetol. 23 (1), 358. 10.1186/s12933-024-02458-x 39395983 PMC11471023

[B109] Rena G. Hardie D. G. Pearson E. R. (2017). The mechanisms of action of metformin. Diabetologia 60 (9), 1577–1585. 10.1007/s00125-017-4342-z 28776086 PMC5552828

[B110] Rossino M. G. Casini G. (2019). Nutraceuticals for the treatment of diabetic retinopathy. Nutrients 11 (4), 771. 10.3390/nu11040771 30987058 PMC6520779

[B111] Ryuk J. A. Mu L. Cao S. Ko B.-S. Park S. (2017). Efficacy and safety of Gegen Qinlian decoction for normalizing hyperglycemia in diabetic patients: a systematic review and meta-analysis of randomized clinical trials. Complement. Ther. Med. 33, 6–13. 10.1016/j.ctim.2017.05.004 28735827

[B112] Sabry K. Jamshidi Z. Emami S. A. Sahebka A. (2024). Potential therapeutic effects of baicalin and baicalein. Avicenna J. Phytomedicine 14 (1), 23–49. 10.22038/AJP.2023.22307 38948180 PMC11210699

[B113] Samuel V. T. Shulman G. I. (2012). Mechanisms for insulin resistance: common threads and missing links. Cell 148 (5), 852–871. 10.1016/j.cell.2012.02.017 22385956 PMC3294420

[B114] Schultze S. M. Hemmings B. A. Niessen M. Tschopp O. (2012). PI3K/AKT, MAPK and AMPK signaling: protein kinases in glucose homeostasis. Expert Rev. Mol. Med. 14, e1. 10.1017/S1462399411002109 22233681

[B115] Shao L. Li M. Zhang B. Chang P. (2020). Bacterial dysbiosis incites Th17 cell revolt in irradiated gut. Biomed. Pharmacother. 131, 110674. 10.1016/j.biopha.2020.110674 32866810

[B116] Shao M. Lu Y. Xiang H. Wang J. Ji G. Wu T. (2022). Application of metabolomics in the diagnosis of non-alcoholic fatty liver disease and the treatment of traditional Chinese medicine. Front. Pharmacol. 13, 971561. 10.3389/fphar.2022.971561 36091827 PMC9453477

[B117] Shen J. Cheng J. Zhu S. Zhao J. Ye Q. Xu Y. (2019). Regulating effect of baicalin on IKK/IKB/NF-kB signaling pathway and apoptosis-related proteins in rats with ulcerative colitis. Int. Immunopharmacol. 73, 193–200. 10.1016/j.intimp.2019.04.052 31103874

[B118] Shen J. Ma H. Wang C. (2021). Triptolide improves myocardial fibrosis in rats through inhibition of nuclear factor kappa B and NLR family pyrin domain containing 3 inflammasome pathway. Korean J. Physiol. Pharmacol. 25 (6), 533–543. 10.4196/kjpp.2021.25.6.533 34697264 PMC8552823

[B119] Shi R. Wang Y. An X. Ma J. Wu T. Yu X. (2019). Efficacy of Co-administration of Liuwei Dihuang pills and Ginkgo Biloba tablets on albuminuria in type 2 diabetes: a 24-Month, multicenter, double-blind, placebo-controlled, randomized clinical trial. Front. Endocrinol. (Lausanne) 10, 100. 10.3389/fendo.2019.00100 30873118 PMC6402447

[B120] Shi X. Q. Chen G. Tan J. Q. Li Z. Chen S. M. He J. H. (2022). Total alkaloid fraction of Leonurus japonicus Houtt. Promotes angiogenesis and wound healing through SRC/MEK/ERK signaling pathway. J. Ethnopharmacol. 295, 115396. 10.1016/j.jep.2022.115396 35598796

[B121] Sougiannis A. T. Enos R. T. VanderVeen B. N. Velazquez K. T. Kelly B. McDonald S. (2021). Safety of natural anthraquinone emodin: an assessment in mice. BMC Pharmacol. Toxicol. 22, 9. 10.1186/s40360-021-00474-1 33509280 PMC7845031

[B122] Tan Y. Liu S. Huang M. Cheng H. Xu B. Luo H. (2024). Efficacy and safety of Gegen Qinlian decoction in the treatment of type II diabetes mellitus: a systematic review and meta-analysis of randomized clinical trials. Front. Endocrinol. (Lausanne) 14, 1316269. 10.3389/fendo.2023.1316269 38344688 PMC10858613

[B123] Tervaert T. W. Mooyaart A. L. Amann K. Cohen A. H. Cook H. T. Drachenberg C. B. (2010). Pathologic classification of diabetic nephropathy. J. Am. Soc. Nephrol. 21 (4), 556–563. 10.1681/ASN.2010010010 20167701

[B124] Thaler J. P. Yi C. X. Schur E. A. Guyenet S. J. Hwang B. H. Dietrich M. O. (2012). Obesity is associated with hypothalamic injury in rodents and humans. J. Clin. Invest. 122 (1), 153–162. 10.1172/JCI59660 22201683 PMC3248304

[B125] Tian J. Jin D. Bao Q. Ding Q. Zhang H. Gao Z. (2019). Evidence and potential mechanisms of traditional Chinese medicine for the treatment of type 2 diabetes: a systematic review and meta-analysis. Diabetes Obes. Metab. 21 (8), 1801–1816. 10.1111/dom.13760 31050124

[B126] Tian J. Zhao Y. Wang L. Li L. (2021). Role of TLR4/MyD88/NF-kappaB signaling in heart and liver-related complications in a rat model of type 2 diabetes mellitus. J. Int. Med. Res. 49 (3), 300060521997590. 10.1177/0300060521997590 33787393 PMC8020098

[B127] Tonks K. T. Ng Y. Miller S. Coster A. C. Samocha-Bonet D. Iseli T. J. (2013). Impaired Akt phosphorylation in insulin-resistant human muscle is accompanied by selective and heterogeneous downstream defects. Diabetologia 56 (4), 875–885. 10.1007/s00125-012-2811-y 23344726

[B128] Trabelsi M. S. Daoudi M. Prawitt J. Ducastel S. Touche V. Sayin S. I. (2015). Farnesoid X receptor inhibits glucagon-like peptide-1 production by enteroendocrine L cells. Nat. Commun. 6, 7629. 10.1038/ncomms8629 26134028 PMC4579574

[B129] Tracey K. J. (2002). The inflammatory reflex. Nature 420 (6917), 853–859. 10.1038/nature01321 12490958

[B130] Vandanmagsar B. Youm Y.-H. Ravussin A. Galgani J. E. Stadler K. Mynatt R. L. (2011). The NLRP3 inflammasome instigates obesity-induced inflammation and insulin resistance. Nat. Med. 17, 179–188. 10.1038/nm.2279 21217695 PMC3076025

[B131] Wang A. Shi X. Yu R. Qiao B. Yang R. Xu C. (2021). The P2X7 receptor is involved in diabetic neuropathic pain hypersensitivity mediated by TRPV1 in the rat dorsal root ganglion. Front. Mol. Neurosci. 14, 663649. 10.3389/fnmol.2021.663649 34163328 PMC8215290

[B135] Wang D. Wang W. Liu T. Gong J. Zou X. Dong H. (2022). Berberine ameliorates glucose metabolism in diabetic rats through the alpha7 nicotinic Acetylcholine receptor-related cholinergic anti-inflammatory pathway. Planta Med. 88 (1), 33–42. 10.1055/a-1385-8015 33682914

[B132] Wang H. Yu M. Ochani M. Amella C. A. Tanovic M. Susarla S. (2003). Nicotinic acetylcholine receptor alpha7 subunit is an essential regulator of inflammation. Nature 421 (6921), 384–388. 10.1038/nature01339 12508119

[B133] Wang Y. Shou J.-W. Li X.-Y. Zhao Z.-X. Fu J. He C.-Y. (2017). Berberine-induced bioactive metabolites of the gut microbiota improve energy metabolism. Metabolism 70, 72–84. 10.1016/j.metabol.2017.02.003 28403947

[B134] Wang S. Chen H. Zheng Y. Li Z. Cui B. Zhao P. (2020). Transcriptomics- and metabolomics-based integration analyses revealed the potential pharmacological effects and functional pattern of *in vivo* Radix Paeoniae Alba administration. Chin. Med. 15, 52. 10.1186/s13020-020-00330-0 32489401 PMC7245909

[B172] Wang S. Chen H. Zheng Y. (2022). Regulation of transient receptor potential channels by traditional Chinese medicine. Front. pharmacol. 13, 1039412. 10.3389/fphar.2022.1039412 36313301 PMC9606675

[B136] Wang Y. Qi F. Li M. Xu Y. Dong L. Cai P. (2025). Traditional Chinese medicine modulates hypothalamic neuropeptides for appetite regulation: a comprehensive review. Biosci. Trends. 19 (3), 281–295. 10.5582/bst.2025.01087 40518285

[B137] Wang F. Liu J. C. Zhou R. J. Zhao X. Liu M. Ye H. (2017). Apigenin protects against alcohol-induced liver injury in mice by regulating hepatic CYP2E1-mediated oxidative stress and PPARalpha-mediated lipogenic gene expression. Chem. Biol. Interact. 275, 171–177. 10.1016/j.cbi.2017.08.006 28803762

[B138] Wang L. Peng W. Zhao Z. Zhang M. Shi Z. Song Z. (2021). Prevalence and treatment of diabetes in China, 2013-2018. JAMA 326 (24), 2498–2506. 10.1001/jama.2021.22208 34962526 PMC8715349

[B139] Wang M. Wang T. Gu F. (2024). Efficacy of Huanglian jiedu decoction for type 2 diabetes mellitus: a systematic review and meta-analysis. Complement. Med. Res. 31 (2), 187–200. 10.1159/000536453 38286111

[B140] Wang X. Pei Z. Hao T. Ariben J. Li S. He W. (2022). Prognostic analysis and validation of diagnostic marker genes in patients with osteoporosis. Front. Immunol. 13, 987937. 10.3389/fimmu.2022.987937 36311708 PMC9610549

[B141] Wang Y. Yu J. Chen B. Jin W. Wang M. Chen X. (2024). Bile acids as a key target: traditional Chinese medicine for precision management of insulin resistance in type 2 diabetes mellitus through the gut microbiota-bile acids axis. Front. Endocrinol. 15, 1481270. 10.3389/fendo.2024.1481270 39720247 PMC11666381

[B142] Wen C. Q. Zou J. Li J. X. Wang F. J. Ge H. T. (2025). Integrating proteomics and network pharmacology to explore the relevant mechanism of Huangkui capsule in the treatment of chronic glomerulonephritis. Front. Pharmacol. 16, 1560420. 10.3389/fphar.2025.1560420 40356949 PMC12067316

[B143] Weng H. Chen J. Ou A. Lao Y. (2022). Leveraging representation learning for the construction and application of a knowledge graph for traditional Chinese medicine: framework development study. JMIR Med. Inf. 10 (9), e38414. 10.2196/38414 36053574 PMC9482071

[B144] Wesemann D. R. (2022). Omicron's message on vaccines: boosting begets breadth. Cell 185 (3), 411–413. 10.1016/j.cell.2022.01.006 35065712 PMC8758338

[B145] Wolf R. C. Gron G. Sambataro F. Vasic N. Wolf N. D. Thomann P. A. (2011). Magnetic resonance perfusion imaging of resting-state cerebral blood flow in preclinical Huntington's disease. J. Cereb. Blood. Flow. Metab. 31 (9), 1908–1918. 10.1038/jcbfm.2011.60 21559028 PMC3185882

[B146] Wong W. Lam C. L. K. Wong V. T. Yang Z. M. Ziea E. T. C. Kwan A. K. L. (2013). Validation of the constitution in Chinese medicine questionnaire: does the traditional Chinese medicine concept of body constitution exist? Evid. Based Complement. Altern. Med. 2013, 481491. 10.1155/2013/481491 23710222 PMC3655622

[B147] Wong A. Bhuiyan M. Rothman J. Drew K. Pourrezaei K. Sun D. (2023). Near infrared spectroscopy detection of hemispheric cerebral ischemia following middle cerebral artery occlusion in rats. Neurochem. Int. 162, 105460. 10.1016/j.neuint.2022.105460 36455748 PMC10263189

[B148] Wu L. Wang Y. Li Z. Zhang B. Cheng Y. Fan X. (2014). Identifying roles of “Jun-Chen-Zuo-Shi” component herbs of QiShenYiQi formula in treating acute myocardial ischemia by network pharmacology. Chin. Med. 9, 24. 10.1186/1749-8546-9-24 25342960 PMC4196468

[B149] Wu W. Meng T. Han L. Jin F. Han P. Zhou Y. (2025). Bridging traditional Chinese medicine and Alzheimer's disease: the pivotal role of gut microbiota multi-target therapeutic mechanisms. Front. Pharmacol. 16, 1630205. 10.3389/fphar.2025.1630205 40657642 PMC12245918

[B150] Xi C. Peng S. Wu Z. Zhou Q. Zhou J. (2017). Toxicity of triptolide and the molecular mechanisms involved. Biomed. Pharmacother. 90, 531–541. 10.1016/j.biopha.2017.04.003 28402922

[B151] Xie Z. Jiang H. Liu W. Zhang X. Chen D. Sun S. (2020). The triterpenoid sapogenin (2alpha-OH-Protopanoxadiol) ameliorates metabolic syndrome via the intestinal FXR/GLP-1 axis through gut microbiota remodeling. Cell Death Dis. 11 (9), 770. 10.1038/s41419-020-02974-0 32943612 PMC7499306

[B152] Xie Y. K. Luo H. Zhang S. X. Chen X. Y. Guo R. Qiu X. Y. (2022). GPR177 in A-fiber sensory neurons drives diabetic neuropathic pain via WNT-mediated TRPV1 activation. Sci. Transl. Med. 14 (639), eabh2557. 10.1126/scitranslmed.abh2557 35385340

[B153] Xu Y. Huang J. Wang N. Tan H. Y. Zhang C. Li S. (2021). Network Pharmacology-Based Analysis and Experimental Exploration of Antidiabetic Mechanisms of Gegen Qinlian Decoction. Front. Pharmacol. 12, 649606. 10.3389/fphar.2021.649606 34381354 PMC8350346

[B181] Xue C. Chen K. Gao Z. Bao T. Dong L. Zhao L. (2023). Common mechanisms underlying diabetic vascular complications: focus on the interaction of metabolic disorders, immuno-inflammation, and endothelial dysfunction. Cell Commun. Signal. 21 (1), 298. 10.1186/s12964-022-01016-w 37904236 PMC10614351

[B154] Yan Z. Wang H. Li J. (2022). Regulation of transient receptor potential channels by traditional Chinese medicine. Front. Pharmacol. 13, 1039412. 10.3389/fphar.2022.1039412 36313301 PMC9606675

[B155] Yang Y. Zhao B. Wang Y. Lan H. Liu X. Hu Y. (2025). Diabetic neuropathy: cutting-edge research and future directions. Signal Transduct. Target. Ther. 10, 132. 10.1038/s41392-025-02175-1 40274830 PMC12022100

[B156] Yao J. Pan J. Jiang Q. Wang H. Zhao Y. (2024). Baicalein inhibits NLRP3 inflammasome activation and mitigates placental inflammation and oxidative stress in gestational diabetes mellitus. Open Life Sci. 19 (1), 20220966. 10.1515/biol-2022-0966 39759105 PMC11699560

[B157] Yi Z. Y. Chen L. Wang Y. He D. Zhao D. Zhang S. H. (2022). The potential mechanism of liu-wei-di-huang Pills in treatment of type 2 diabetic mellitus: from gut microbiota to short-chain fatty acids metabolism. Acta Diabetol. 59, 1295–1308. 10.1007/s00592-022-01922-y 35857109

[B158] Yin Y. Qu H. Yang Q. Fang Z. Gao R. (2021). Astragaloside IV alleviates Schwann cell injury in diabetic peripheral neuropathy by regulating microRNA-155-mediated autophagy. Phytomedicine 92, 153749. 10.1016/j.phymed.2021.153749 34601220

[B159] You H. Zhang T. Feng W. Gai Y. (2017). Association of TCM body constitution with insulin resistance and risk of diabetes in impaired glucose regulation patients. BMC Complement. Altern. Med. 17, 459. 10.1186/s12906-017-1964-0 28893239 PMC5594579

[B160] Zhang Y. Gu Y. Ren H. Wang S. Zhong H. Zhao X. (2020). Gut microbiome-related effects of berberine and probiotics on type 2 diabetes (the PREMOTE study). Nat. Commun. 11, 5015. 10.1038/s41467-020-18414-8 33024120 PMC7538905

[B161] Zhang D. Jian Y. P. Zhang Y. N. Li Y. Gu L. T. Sun H. H. (2023). Short-chain fatty acids in diseases. Cell Commun. Signal. 21 (1), 212. 10.1186/s12964-023-01219-9 37596634 PMC10436623

[B162] Zhang Y. Shi M. Peng D. Chen W. Ma Y. Song W. (2024). QiMing granules for diabetic retinopathy: a systematic review and meta-analysis of randomized controlled trials. Front. Pharmacol. 15, 1429071. 10.3389/fphar.2024.1429071 39239647 PMC11374745

[B163] Zhang Y. Liu S. Cao D. Zhao M. Lu H. Wang P. (2025). Rg1 improves Alzheimer's disease by regulating mitochondrial dynamics mediated by the AMPK/Drp1 signaling pathway. J. Ethnopharmacol. 340, 119285. 10.1016/j.jep.2024.119285 39733799

[B175] Zhao P. Zhang X. Gong Y. Li W. Wu Z. Tang Y. (2022). Investigation of the mechanism of Shen Qi Wan prescription in the treatment of T2DM via network pharmacology and molecular docking. In Silico Pharmacol. 10 (1), 9. 10.1007/s40203-022-00124-2 35673584 PMC9167366

[B164] Zhao T. Lun S. Yan M. Park J. Wang S. Chen C. (2024). 6,7-Dimethoxycoumarin, Gardenoside and Rhein combination improves non-alcoholic fatty liver disease in rats. J. Ethnopharmacol. 322, 117646. 10.1016/j.jep.2023.117646 38135236

[B165] Zheng J. Liao Y. Xu Y. Mo Z. (2022). Icariin attenuates ischaemic stroke through suppressing inflammation mediated by endoplasmic reticulum stress signaling pathway in rats. Clin. Exp. Pharmacol. Physiol. 49 (7), 719–730. 10.1111/1440-1681.13645 35451526

[B166] Zheng Y. Xu L. Dong N. Li F. (2022). NLRP3 inflammasome: the rising star in cardiovascular diseases. Front. Cardiovasc Med. 9, 927061. 10.3389/fcvm.2022.927061 36204568 PMC9530053

[B167] Zhou R. Tardivel A. Thorens B. Choi I. Tschopp J. (2010). Thioredoxin-interacting protein links oxidative stress to inflammasome activation. Nat. Immunol. 11 (2), 136–140. 10.1038/ni.1831 20023662

[B168] Zhou X. Seto S. W. Chang D. Kiat H. Razmovski-Naumovski V. Chan K. (2016). Synergistic effects of Chinese herbal medicine: a comprehensive review of methodology and current research. Front. Pharmacol. 7, 201. 10.3389/fphar.2016.00201 27462269 PMC4940614

[B169] Zhuang Y. Yu L. Jiang N. Ge Y. (2025). TCM-KLLaMA: intelligent generation model for Traditional Chinese Medicine Prescriptions based on knowledge graph and large language model. Comput. Biol. Med. 189, 109887. 10.1016/j.compbiomed.2025.109887 40056842

[B170] Zou F. Mao X. Q. Wang N. Liu J. Ou-Yang J. P. (2009). Astragalus polysaccharides alleviates glucose toxicity and restores glucose homeostasis in diabetic states via activation of AMPK. Acta Pharmacol. Sin. 30 (12), 1607–1615. 10.1038/aps.2009.168 19960007 PMC4007496

