# Supplementary material for: Pharmacological advances in multi-targeted strategies for type 2 diabetes mellitus: a systematic perspective based on traditional Chinese medicine
Source: Front Pharmacol. 2026 Feb 20;16:1732134. doi: 10.3389/fphar.2025.1732134 (PMC12963220; doi:10.3389/fphar.2025.1732134)
Supplement: Supplementary file 1 [file Supplementaryfile1.docx]

Supplementary Tables

Table S1. Preclinical pharmacology evidence matrix (including extract type and characterization/QC). Table S1 is presented in two parts (S1A and S1B) to improve readability on the page.

| Indication/axis | Intervention | Extract type | Characterization (QC/purity) | Model (in vitro/in vivo) | Dose/MAC | Controls | Duration | Reference |
| --- | --- | --- | --- | --- | --- | --- | --- | --- |
| Insulin sensitivity | Astragalus polysaccharides | Extract fraction (polysaccharide; as stated) | Composition/marker QC NR | Preclinical (as cited) | NR | NR | NR | Kearney et al., 2021 |
| Insulin resistance | Puerarin | Isolated plant metabolite (as stated) | Purity/identity verification NR | Preclinical (as cited) | NR | NR | NR | Huang et al., 2012 |
| Energy metabolism | Berberine | Isolated plant metabolite (as stated) | Purity/identity verification NR | Preclinical (as cited) | NR | NR | NR | Lee et al., 2006; Wang et al., 2024 |
| Gut hormone signaling | Gardenoside | Isolated plant metabolite (as stated) | Purity/identity verification NR | Preclinical (as cited) | NR | NR | NR | Wang et al., 2022 |
| Bile-acid signaling | Rhein | Isolated plant metabolite (as stated) | Purity/identity verification NR | Preclinical (as cited) | NR | NR | NR | Hu et al., 2024; Zhao et al., 2024 |
| DN (renal fibrosis) | Huanglian Jiedu Decoction | Multi-botanical preparation (as stated) | Marker/batch QC NR; chemical profiling NR | In vivo: STZ diabetic rat | NR | NR | NR | Cai et al., 2025 |
| DR (retinal inflammation) | Baicalin | Isolated plant metabolite (as stated) | Purity/identity verification NR | In vitro: ARPE-19 high glucose | NR | NR | NR | Sabry et al., 2024 |
| DR (neovascularization) | Baicalin | Isolated plant metabolite (as stated) | Purity/identity verification NR | In vivo: STZ diabetic mouse | 50 mg/kg oral | Vehicle | NR | Gong et al., 2025 |
| Neuroprotection exemplar | Ginsenoside Rg1 | Isolated plant metabolite (as stated) | Purity/identity verification NR | In vivo: APP/PS1 mouse (as cited) | NR | Model group | NR | Zhang et al., 2025; Nie et al., 2017 |
| Oxidative stress (cardiac) | Icariin | Isolated plant metabolite (as stated) | Purity/identity verification NR | Preclinical (as cited) | NR | NR | NR | Li et al., 2022; Zheng et al., 2022 |
| Cardiomyocytes (HG injury) | Tanshinone IIA | Isolated plant metabolite (as stated) | Purity/identity verification NR | In vitro: H9c2 high glucose | IC50 12.6 μM (cell viability) | NR | NR | Ji et al., 2018 |
| Cardiac apoptosis | Tanshinone IIA | Isolated plant metabolite (as stated) | Purity/identity verification NR | In vivo: STZ diabetic mouse | NR | NR | NR | Moura et al., 2021 |
| Neuroinflammation | Gastrodin | Isolated plant metabolite (as stated) | Purity/identity verification NR | Preclinical (as cited) | NR | NR | NR | Wong et al., 2023 |
| Inflammasome | Triptolide | Isolated plant metabolite (as stated) | Purity/identity verification NR | Preclinical (as cited) | NR | NR | NR | Ding et al., 2024 |

Table S1 (continued). Part B: Mechanistic/endpoints and ConPhyMP reporting fields.

| Indication/axis | Intervention | Key pharmacology/mechanism | Key endpoints | Main findings | Reporting gaps | Botanical identity (validated species/part/voucher) | Preparation processing (for formulas; extraction/ratio) | Reference |
| --- | --- | --- | --- | --- | --- | --- | --- | --- |
| Insulin sensitivity | Astragalus polysaccharides | Facilitates PI3K–IRS→Akt activation | Insulin signaling readouts; glucose uptake | Improved insulin signaling in preclinical contexts | Taxonomy/QC, dose, and comparator details NR | NR (not reported) / see Supplementary Table Sx | NR (not reported) / see Supplementary Table Sx | Kearney et al., 2021 |
| Insulin resistance | Puerarin | JNK inhibition protects IRS-1/2 signaling | IRS-1/2 integrity; Akt signaling; glycemia | Mechanistic support for improved insulin signaling | Dose/route/duration and controls NR | NR (not reported) / see Supplementary Table Sx | NR (not reported) / see Supplementary Table Sx | Huang et al., 2012 |
| Energy metabolism | Berberine | Activates AMPK; promotes GLUT4 translocation; may modulate BA/TGR5–GLP-1 | AMPK phosphorylation; GLUT4 translocation; GLP-1; glycemia | Preclinical evidence for improved glucose handling | Concentration/dose, PK, and assay counter-screens NR | NR (not reported) / see Supplementary Table Sx | NR (not reported) / see Supplementary Table Sx | Lee et al., 2006; Wang et al., 2024 |
| Gut hormone signaling | Gardenoside | TGR5 agonism; stimulates GLP-1 secretion | GLP-1; insulin sensitivity | Mechanistic support for incretin-mediated benefit | Dose/route and model details NR | NR (not reported) / see Supplementary Table Sx | NR (not reported) / see Supplementary Table Sx | Wang et al., 2022 |
| Bile-acid signaling | Rhein | Epigenetic suppression of CYP7A1; FXR/SHP modulation | BA composition; hepatic inflammation | Mechanistic link to BA remodeling and inflammation control | Dose/PK and endpoints NR | NR (not reported) / see Supplementary Table Sx | NR (not reported) / see Supplementary Table Sx | Hu et al., 2024; Zhao et al., 2024 |
| DN (renal fibrosis) | Huanglian Jiedu Decoction | Anti-fibrotic signaling (TGF-β1/collagen I) | 24-h urine protein; renal TGF-β1 & collagen I (IHC) | 24-h urine protein ~−35%; IHC score TGF-β1/collagen I ~−40% | Need dose, duration, QC markers, taxonomic validation, and comparator arms | NR (not reported) / see Supplementary Table Sx | NR (not reported) / see Supplementary Table Sx | Cai et al., 2025 |
| DR (retinal inflammation) | Baicalin | TLR4/NF-κB inhibition; anti-VEGF | VEGF; NF-κB p65 protein | VEGF −52%; p65 protein −37% | Need concentration, exposure time, assay details, and orthogonal validation | NR (not reported) / see Supplementary Table Sx | NR (not reported) / see Supplementary Table Sx | Sabry et al., 2024 |
| DR (neovascularization) | Baicalin | Anti-angiogenic via VEGFR2-related signaling (as cited) | Retinal neovascularization area | Neovascularization area −33% | Need duration, blinding/randomization, and positive control reporting | NR (not reported) / see Supplementary Table Sx | NR (not reported) / see Supplementary Table Sx | Gong et al., 2025 |
| Neuroprotection exemplar | Ginsenoside Rg1 | PI3K/Akt–BDNF activation | Morris water maze; hippocampal BDNF | Escape latency −41.3%; hippocampal BDNF ×2.4; ↑Akt phosphorylation | Not a diabetes model; requires T2DM/DCD-specific validation and dosing details | NR (not reported) / see Supplementary Table Sx | NR (not reported) / see Supplementary Table Sx | Zhang et al., 2025; Nie et al., 2017 |
| Oxidative stress (cardiac) | Icariin | NRF2 activation; ↓ERS (IRE1α–XBP1) | SOD2; MDA; cardiac function | Reported antioxidant and functional improvements (quantitative details not summarized) | Need numeric outcomes, dose, and model details | NR (not reported) / see Supplementary Table Sx | NR (not reported) / see Supplementary Table Sx | Li et al., 2022; Zheng et al., 2022 |
| Cardiomyocytes (HG injury) | Tanshinone IIA | Antioxidant; supports NRF2/HO-1 axis (as cited) | ROS; SOD2; MDA | SOD2 ×1.9; MDA −46.2%; ROS ↓~60% (as described) | Need exposure time, assay method, and fluorescence/redox interference controls | NR (not reported) / see Supplementary Table Sx | NR (not reported) / see Supplementary Table Sx | Ji et al., 2018 |
| Cardiac apoptosis | Tanshinone IIA | Antioxidant; anti-apoptotic | Cardiac TUNEL assay | TUNEL+ cells −41% | Need dose/route/duration and functional endpoints (echo) | NR (not reported) / see Supplementary Table Sx | NR (not reported) / see Supplementary Table Sx | Moura et al., 2021 |
| Neuroinflammation | Gastrodin | TLR4/NF-κB inhibition | CNS inflammatory markers; cognition | Neuroprotective effects in diabetes-relevant contexts (details NR) | Need quantitative outcomes and dose details | NR (not reported) / see Supplementary Table Sx | NR (not reported) / see Supplementary Table Sx | Wong et al., 2023 |
| Inflammasome | Triptolide | Disrupts NEK7-mediated NLRP3 assembly | Caspase-1; IL-1β/IL-18; NLRP3 readouts | Inflammasome suppression reported (details NR) | Safety window and toxicity reporting required | NR (not reported) / see Supplementary Table Sx | NR (not reported) / see Supplementary Table Sx | Ding et al., 2024 |

Table note: Some phytochemicals may show assay-interference liabilities (including PAINS); support mechanisms with orthogonal validation and appropriate counter-screens.

Abbreviations: DN, diabetic nephropathy; DR, diabetic retinopathy; DCM, diabetic cardiomyopathy; DCD, diabetes-associated cognitive decline; STZ, streptozotocin; MAC, minimal active concentration; NR, not reported.

Table S2. Clinical evidence summary (including intervention standardization/QC).

| **Study** | **Population** | **Design** | **N** | **Intervention (dose/form)** | **Standardization/QC** | **Comparator/background** | **Follow-up** | **Key endpoints** | **Main results** | **Safety** | **Limitations/notes** | Composition/processing (for multi-botanical; extraction/QC) |
| --- | --- | --- | --- | --- | --- | --- | --- | --- | --- | --- | --- | --- |
| Hu et al., 2021 | Adults with T2DM | Systematic review & meta-analysis (RCTs) | 9 studies; HbA1c pooled n=461 (6 studies) | HLJDD adjunct; formulation/dose varied | Varied; batch/marker QC NR | Conventional therapy controls (varied) | Varied across RCTs | HbA1c; FBG; 2hPG; lipids; AEs | HbA1c MD −1.08% (95% CI −1.45 to −0.70) | AE reporting inconsistent; no higher AE rate vs controls reported | Heterogeneous formulations; variable trial quality | NR (not reported) / see Supplementary Table Sx |
| (as cited in MS) | Adults with T2DM | Clinical evidence summarized in MS | NR | HLJDD + metformin | NR (batch/markers/dosage form details) | Metformin (standard) | NR | HbA1c; insulin resistance | MS reports HbA1c decrease ~1.3% and improved insulin resistance | NR | Please add full trial citation and design details | NR (not reported) / see Supplementary Table Sx |
| Zhang et al., 2024 | Diabetic retinopathy | Systematic review & meta-analysis (RCTs) | See source meta-analysis | Qiming Granules, often adjunctive | Dosage form stated; batch/marker QC NR | Controls varied; some trials with anti-VEGF regimens | Varied across RCTs | VA; macular edema; injection frequency; AEs | MS notes reduced intravitreal injection frequency when combined with anti-VEGF | NR | Provide effect sizes and quality grading for injection-frequency endpoint | NR (not reported) / see Supplementary Table Sx |
| (as cited in MS) | T2DM + DN | Multicenter RCT (cited in MS) | 268 (as stated in MS) | Huang Kui Capsules (Abelmoschus manihot) | Dosage form stated; batch/marker QC NR | Standard-of-care background therapy (NR) | NR | eGFR; proteinuria; safety | MS reports stabilized eGFR and reduced proteinuria | NR | Need full citation, dose/duration, randomization/blinding, and AE reporting | NR (not reported) / see Supplementary Table Sx |

Table note: Interpret clinical effects with attention to study quality, intervention standardization/QC, and concomitant therapies; prioritize clinically meaningful endpoints and transparent safety reporting.

**Table notes (added to address GA-online/ConPhyMP reporting requirements):**

**† Botanical source species are taxonomically validated; full species names with authorities and family, plant part, and voucher information (if reported) are provided in Supplementary Table Sx; items not reported in the primary study are recorded as NR.
‡ For multi-botanical preparations, complete composition (all component drugs with validated species + authority + family), processing/preparation (e.g., decoction/granule/pill; extraction/solvent and ratio), and chemical characterization/QC markers are reported in Supplementary Table Sx.**

**‡ For multi-botanical interventions in clinical studies, complete composition (validated species + authority + family), dosage form (decoction/granule/pill/patent medicine), manufacturer/batch (if available), processing/extraction details, and chemical characterization/QC marker information are compiled in Supplementary Table Sx; unreported items are recorded as NR.**
