# Supplementary figures and images for: Pharmacological advances in multi-targeted strategies for type 2 diabetes mellitus: a systematic perspective based on traditional Chinese medicine
Source: Front Pharmacol. 2026 Feb 20;16:1732134. doi: 10.3389/fphar.2025.1732134 (PMC12963220; doi:10.3389/fphar.2025.1732134)

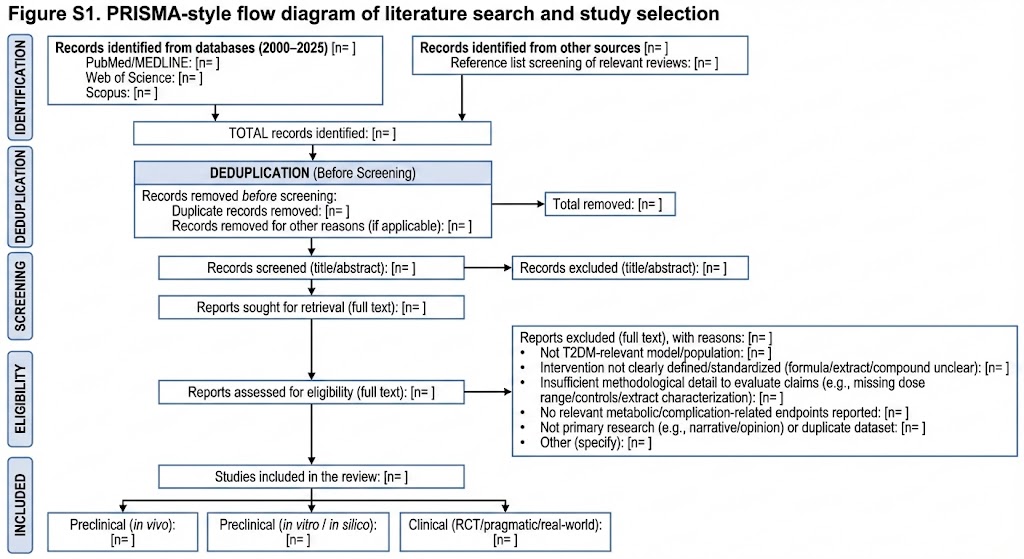

Supplement: Supplementary file 2 [file Image1.jpeg]
