# Supplementary material for: Pharmacological advances in multi-targeted strategies for type 2 diabetes mellitus: a systematic perspective based on traditional Chinese medicine
Source: Front Pharmacol. 2026 Feb 20;16:1732134. doi: 10.3389/fphar.2025.1732134 (PMC12963220; doi:10.3389/fphar.2025.1732134)
Supplement: Supplementary file 3 [file Supplementaryfile2.docx]

**Supplementary File S2**

**GA-online Best Practice Tool (ConPhyMP) – Completed Checklists (Tables 1 and 2A)**

| **Journal (target)** | Frontiers in Pharmacology |
| --- | --- |
| **Manuscript** | Review on TCM interventions in Type 2 Diabetes Mellitus (T2DM) and complications |
| **Date** | 2025-12-16 |

Note: ConPhyMP is designed to improve reproducibility/interpretability of studies using medicinal-plant materials and extracts. As this submission is a review, we do not prepare plant material or extracts. Instead, we report our literature search/selection and perform critical appraisal (evidence tiering; reporting gaps) and summarize study-level details in Supplementary Tables S1–S2. Items that require primary experimental handling are marked N/A.

**ConPhyMP Table 1. Reporting plant material and its initial processing (review-level completion).**

| **Section/Topic** | **Item** | **Checklist item** | **Y/N/N-A** | **Location in manuscript** |
| --- | --- | --- | --- | --- |
| Title & abstract | 1 | Clear title and informative abstract with balanced summary. | Y | Title; Abstract |
| Botanical drug / taxonomy | 2 | Taxonomic authentication info for plant materials (when applicable). | Y (appraisal) | Methods (critical appraisal); Table S1 (taxonomic validity/identity fields, where reported) |
| Extract description | 3 | Extraction/process details (species, processing, extraction) reported in Methods. | N/A | Review article; summarized as reported in Table S1 (extract/exposure/characterization fields) |
| Legal basis | 4 | Compliance with Nagoya/CITES/phytosanitary treaties. | N/A | Not applicable to review |
| Commercial products | 5 | Batch/production date/regulatory status for finished products. | Y (where reported) | Table S2 (standardization/dosage form/limitations) |

**ConPhyMP Table 2A. Analytical methods checklist (extract type A) – review-level completion.**

| **Section/Topic** | **Item** | **Checklist item** | **Y/N/N-A** | **Location in manuscript** |
| --- | --- | --- | --- | --- |
| Extract type | 1 | Pharmacopoeia monograph coverage (where applicable). | N/A | Review article; recorded where available in Table S1 |
| Preferred/main methods | 2 | Markers, certificates/CoA, fingerprinting, ≥2 marker quantification (as applicable). | Y (appraisal) | Methods (critical appraisal); Table S1 (characterization fields) |
| Alternative methods | 3 | Single fingerprinting with ≥3 detection parameters; ≥2 marker quantification (as applicable). | Y (appraisal) | Table S1 (characterization/limitations) |
| Reference standards | 4 | Use/overlay of official reference standards (where applicable). | Y (gap-tracked) | Table S1 (limitations/reporting gaps) |
| Comparability across samples | 5 | Similarity scoring / direct comparisons across extracts/samples (where applicable). | Y (gap-tracked) | Table S1; Discussion (Limitations & research needs) |

**References**

Heinrich M, Jalil B, Abdel-Tawab M, Echeverria J, Kulić Ž, McGaw LJ, et al. (2022). Best Practice in the chemical characterisation of extracts used in pharmacological and toxicological research—The ConPhyMP—Guidelines. Front Pharmacol 13:953205. doi:10.3389/fphar.2022.953205.

GA-online (accessed 2025-12-16). Best Practice in Research – ConPhyMP. https://ga-online.org/best-practice/
